# Depletion of protein kinase STK25 ameliorates renal lipotoxicity and protects against diabetic kidney disease

**DOI:** 10.1172/jci.insight.140483

**Published:** 2020-12-17

**Authors:** Emmelie Cansby, Mara Caputo, Lei Gao, Nagaraj M. Kulkarni, Annika Nerstedt, Marcus Ståhlman, Jan Borén, Rando Porosk, Ursel Soomets, Matteo Pedrelli, Paolo Parini, Hanns-Ulrich Marschall, Jenny Nyström, Brian W. Howell, Margit Mahlapuu

**Affiliations:** 1 Department of Chemistry and Molecular Biology and; 2 Department of Molecular and Clinical Medicine/Wallenberg Laboratory, Institute of Medicine, University of Gothenburg and Sahlgrenska University Hospital, Gothenburg, Sweden.; 3 Department of Biochemistry, Institute of Biomedicine and Translational Medicine, University of Tartu, Estonia.; 4 Department of Laboratory Medicine and; 5 Metabolism Unit, Department of Medicine, Karolinska Institutet, Stockholm, Sweden.; 6 Theme Inflammation and Infection, Karolinska University Hospital, Stockholm, Sweden.; 7 Department of Physiology, Institute of Neuroscience and Physiology, the Sahlgrenska Academy, University of Gothenburg, Gothenburg, Sweden.; 8 Department of Neuroscience and Physiology, State University of New York Upstate Medical University, Syracuse, New York, USA.

**Keywords:** Metabolism, Nephrology, Chronic kidney disease, Diabetes, Fibrosis

## Abstract

Diabetic kidney disease (DKD) is the most common cause of severe renal disease worldwide and the single strongest predictor of mortality in diabetes patients. Kidney steatosis has emerged as a critical trigger in the pathogenesis of DKD; however, the molecular mechanism of renal lipotoxicity remains largely unknown. Our recent studies in genetic mouse models, human cell lines, and well-characterized patient cohorts have identified serine/threonine protein kinase 25 (STK25) as a critical regulator of ectopic lipid storage in several metabolic organs prone to diabetic damage. Here, we demonstrate that overexpression of STK25 aggravates renal lipid accumulation and exacerbates structural and functional kidney injury in a mouse model of DKD. Reciprocally, inhibiting STK25 signaling in mice ameliorates diet-induced renal steatosis and alleviates the development of DKD-associated pathologies. Furthermore, we find that STK25 silencing in human kidney cells protects against lipid deposition, as well as oxidative and endoplasmic reticulum stress. Together, our results suggest that STK25 regulates a critical node governing susceptibility to renal lipotoxicity and that STK25 antagonism could mitigate DKD progression.

## Introduction

Diabetic kidney disease (DKD) occurs in almost one-third of diabetes patients and is one of the most fatal long-term diabetic complications (1). The structural hallmarks of DKD include glomerular mesangial expansion (GME), glomerular basement membrane (GBM) thickening, podocyte loss, tubulointerstitial injury, renal fibrosis, and arteriolar hyalinosis, resulting in a progressive decline in kidney function. Although DKD is the most common cause of end-stage renal disease (ESRD) in the Western world, accounting for nearly 40% of newly diagnosed cases that require dialysis or kidney transplantation (1), its molecular pathogenesis remains incompletely understood.

Hyperglycemia has been suggested as the main underlying cause for the progression of DKD. However, multiple large-scale studies have now demonstrated that intensive glycemic control has only a modest effect on renal endpoints in diabetes (2). Hence, disease mechanisms other than hyperglycemia may be more relevant in DKD (3). Recently, kidney lipotoxicity (i.e., lipid accumulation in glomerular and tubular cells) has emerged as a critical trigger in the pathogenesis of DKD through induction of renal oxidative stress, chronic low-grade inflammation, and extracellular matrix deposition (4–8). Deciphering the molecular mechanisms responsible for excessive renal lipid storage is therefore important to accelerate the design of novel therapeutic strategies against DKD.

In search of novel targets that control ectopic lipid deposition, we recently identified serine/threonine protein kinase 25 (STK25), a member of the sterile 20 kinase superfamily (9), as a critical regulator of lipotoxicity in the context of nutritional stress and obesity. We found that mice with global overexpression of STK25 accumulate excessive fat in the liver, skeletal muscle, pancreas, and vascular wall, which is accompanied by the influx of macrophages and nutritional fibrosis, and the reciprocal phenotype is seen in *Stk25*-KO mice (10–17). Furthermore, we found that STK25 mRNA and protein levels correlate with the severity of hepatic steatosis and lobular inflammation in human liver biopsies, and several common nonlinked SNPs in the human *STK25* gene are associated with altered liver fat (15, 18).

STK25 is highly expressed in human and rodent kidneys (19), but its renal function is unknown. On the basis of our previous findings, which reveal a critical role of STK25 in the control of lipid accumulation, chronic low-grade inflammation, and fibrosis in several metabolic organs prone to diabetic damage, we hypothesized that STK25 is also involved in regulation of renal dysfunction in connection to obesity. Here, we investigated the impact of STK25 signaling on susceptibility to lipotoxicity-mediated progression of DKD using a model of high-fat diet–fed mice that displays renal lesions similar to DKD in patients with metabolic syndrome (20). In addition, we performed in vitro studies using cultured human kidney cells to assess the direct renoprotective role of STK25 inactivation.

## Results

### Overexpression of STK25 in mice aggravates the development of diet-induced DKD.

**Figure 1:**
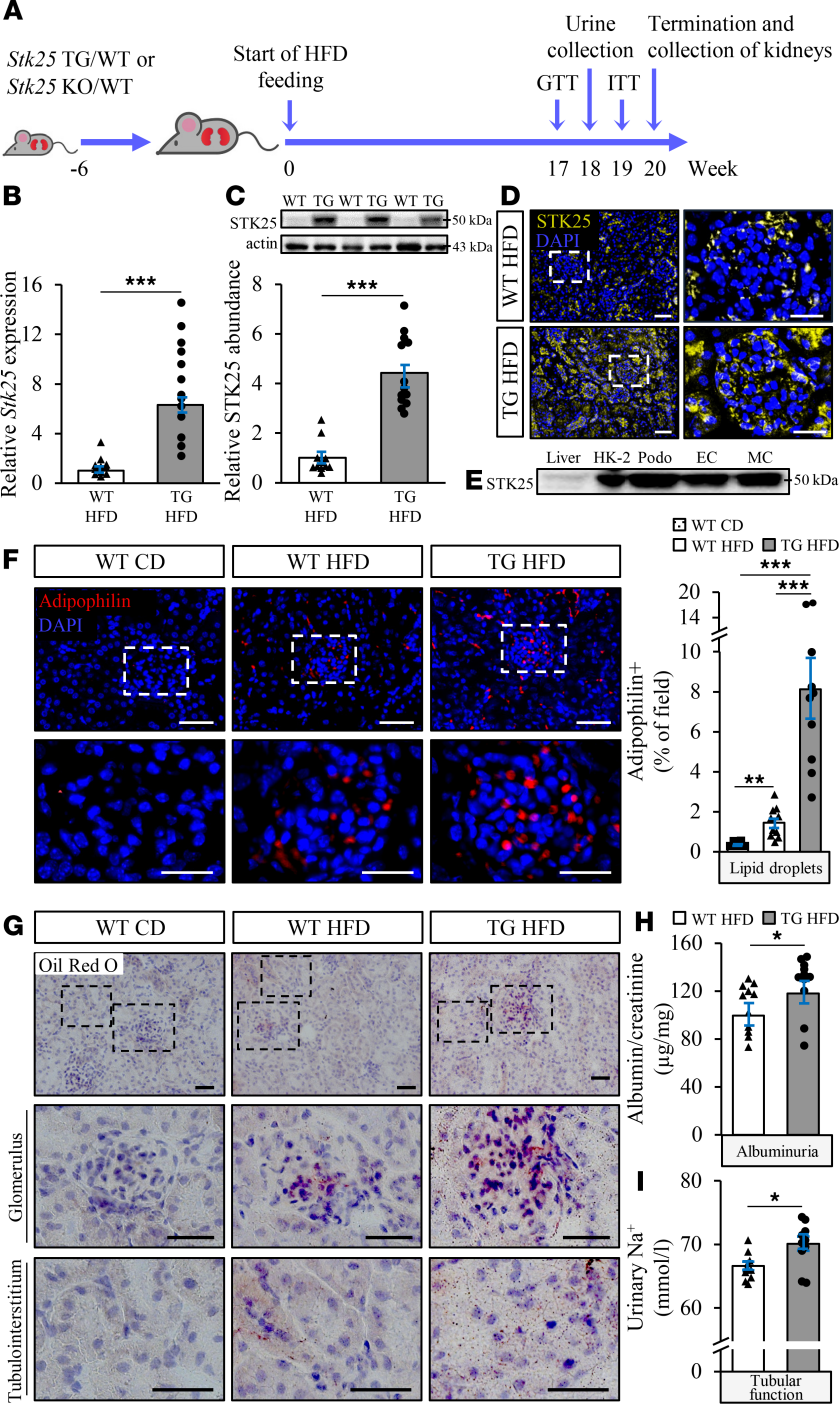
Overexpression of STK25 in mice aggravates high-fat diet–induced renal lipid accumulation and impairs kidney function. (**A**) Schematic presentation of the experimental design of the in vivo study. (**B** and **C**) Renal STK25 mRNA (**B**) and protein (**C**) abundance. Protein levels were analyzed by densitometry; representative Western blots are shown with pan-actin used as a loading control. (**D**) Representative kidney sections processed for immunofluorescence with anti-STK25 antibody (yellow); nuclei stained with DAPI (blue). (**E**) Representative Western blot comparing protein expression of STK25 in kidney cells of human origin; liver is included as a reference. (**F**) Representative kidney sections processed for immunofluorescence with anti-adipophilin antibody (red); nuclei stained with DAPI (blue). Quantification of the staining. (**G**) Representative kidney sections stained with Oil Red O. (**H** and **I**) Measurement of urinary ACR (**H**) and sodium levels (**I**). In **D**, the scale bars at the left and right represent 50 and 25 μm, respectively; in **F**, the scale bars at the top and bottom represent 50 and 25 μm, respectively; in **G**, the scale bars at the top and 2 bottom represent 50 and 25 μm, respectively. Data are mean ± SEM from 8–12 mice per group. CD, chow diet; EC, endothelial cells; HFD, high-fat diet; HK-2, human kidney-2; MC, mesangial cells; Podo, podocytes; TG, transgenic. *P < 0.05, ***P < 0.001 by a 2-tailed Student’s *t* test in **B**, **C**, **H**, and **I**, and 1-way ANOVA followed by a 2-tailed Student’s *t* test in **F**.

Mice fed a high-fat diet during an extended period of time is a common nonclinical model for studies of DKD (6). Here, we analyzed whether overexpression of STK25 in high-fat diet–fed mice exacerbates diet-induced renal lipid accumulation and progression of DKD (Figure 1A). On the high-fat diet, the STK25 mRNA and protein abundance was upregulated 6.3 ± 0.2– and 4.4 ± 0.4–fold, respectively, in whole kidney lysates from transgenic mice compared with WT littermates (Figure 1, B and C). Notably, renal protein levels of STK25 were not affected by the diet (Supplemental Figure 1; supplemental material available online with this article; https://doi.org/10.1172/jci.insight.140483DS1). In kidney samples from WT mice, endogenous STK25 protein could be detected in both glomerular and renal tubular cells, and this expression was augmented in transgenic mice (Figure 1D and Supplemental Figure 2, A and B). Consistent with the broad expression of STK25 throughout mouse kidney, we found that human podocytes, endothelial and mesangial cells, and tubular epithelial cells express high levels of endogenous STK25 protein (Figure 1E).

During the high-fat feeding, lipid droplets visualized by immunofluorescence staining of adipophilin (also known as ADRP or PLIN2), and neutral lipids (triglycerides and cholesterol esters) visualized by Oil Red O staining, accumulated in the kidneys from WT mice (Figure 1, F and G). Importantly, we found aggravated glomerular as well as tubular deposition of lipid droplets and neutral lipids in high-fat diet–fed *Stk25* transgenic mice compared with WT controls (Figure 1, F and G). In parallel, elevated albuminuria (measured as the urinary albumin/creatinine ratio) was observed in *Stk25* transgenic versus WT mice after a dietary challenge, indicating exacerbated glomerular injury with loss of permselectivity (Figure 1H). Higher urinary levels of sodium detected in high-fat diet–fed *Stk25* transgenic mice may also suggest an aggravated tubular injury (Figure 1I). Overexpression of STK25 had no effect on the renin-angiotensin system (Supplemental Figure 3, A and B).

**Figure 2:**
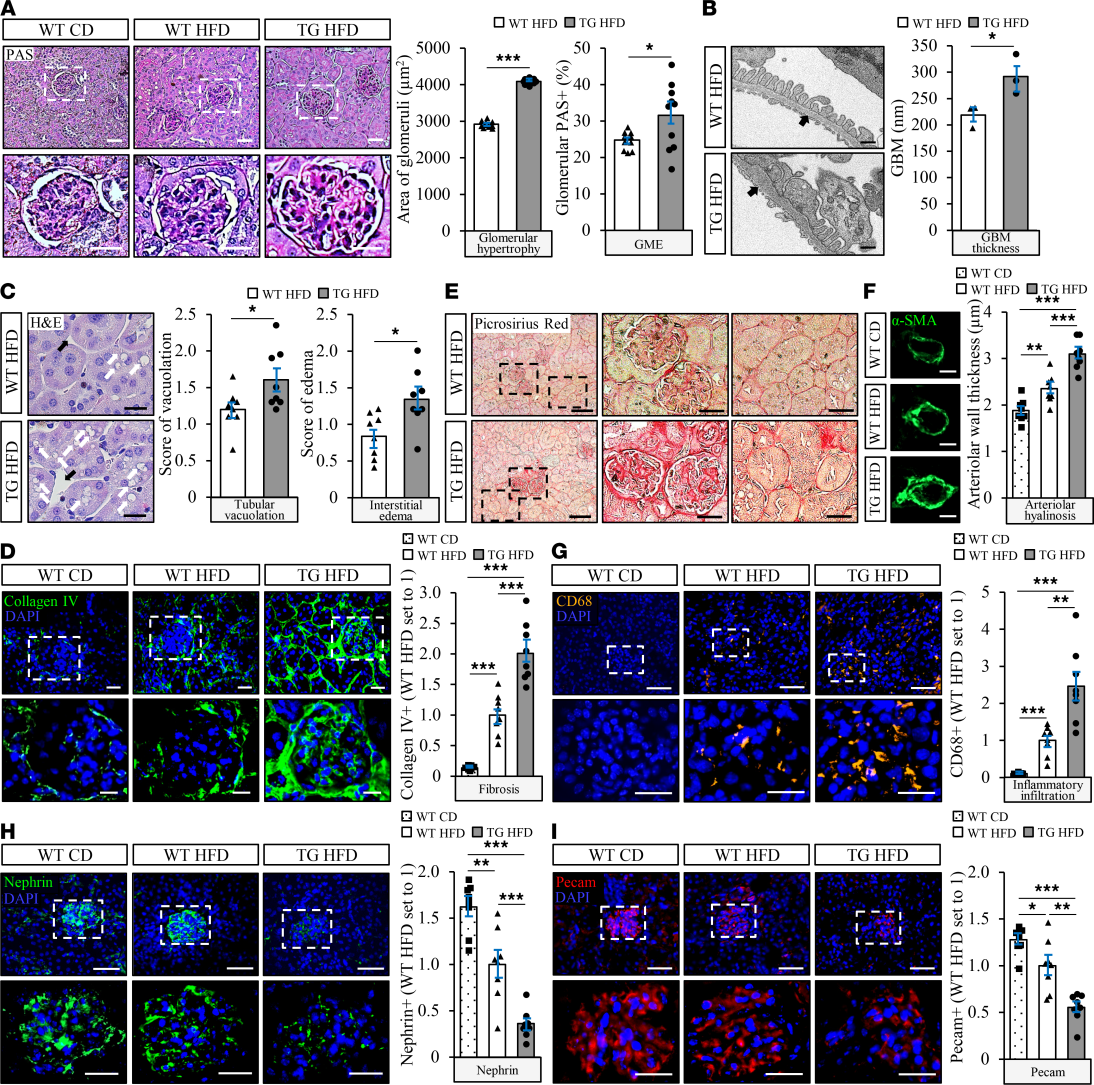
STK25 overexpression in mice exacerbates high-fat diet–induced glomerular mesangial matrix expansion, GBM thickening, tubulointerstitial injury, glomerulosclerosis and tubulointerstitial fibrosis, renal inflammation, and arteriolar hyalinosis, and it impairs the integrity of GFB. (**A**) Representative kidney sections stained with PAS and quantification of glomerular hypertrophy and GME. (**B**) Representative TEM images of the kidney and measurement of the thickness of GBM (black arrows). (**C**) Representative kidney sections stained with H&E and scoring of tubular vacuolation (white arrows) and interstitial edema (black arrows). (**D**, **G**–**I**) Representative kidney sections processed for immunofluorescence with anti–collagen IV (green; **D**), anti-CD68 (yellow; **G**), anti-nephrin (green; **H**), or anti-Pecam (red; **I**) antibodies; nuclei stained with DAPI (blue). Quantification of the staining. (**E**) Representative kidney sections stained with Picrosirius red. (**F**) Representative kidney sections processed for immunofluorescence with anti–α-SMA (green) antibody and quantification of arteriolar wall thickness. In **A**, **D**, and **G**–**I**, the scale bars at the top and bottom represent 50 and 25 μm, respectively; in **B**, the scale bars represent 500 nm; in **C**, the scale bars represent 50 μm; in **E**, the scale bars at the left and middle/right represent 100 and 25 μm, respectively; in **F**, the scale bars represent 10 μm. Data are mean ± SEM from 8–10 mice per group, except in **B**, where *n* = 3 per group. CD, chow diet; HFD, high-fat diet; TG, transgenic. *P < 0.05, **P < 0.01, ***P < 0.001 by a 2-tailed Student’s *t* test in **A**–**C** and 1-way ANOVA followed by a 2-tailed Student’s *t* test in **D** and **F**–**I**.

Kidney sections from *Stk25* transgenic and WT mice collected at the end of the high-fat diet feeding period were analyzed for glomerular and tubular injuries typically seen in DKD. Periodic acid–Schiff (PAS) staining and scanning electron microscopy analysis showed that glomerular hypertrophy and GME were significantly increased in *Stk25* transgenic versus WT kidneys (Figure 2A and Supplemental Figure 4A). A morphometric analysis of the transmission electron microscopy (TEM) images also revealed an impairment in GBM thickness and exacerbated podocyte vacuolation in *Stk25* transgenic kidneys (Figure 2B and Supplemental Figure 4B). Semiquantitative scoring of H&E-stained kidney sections further demonstrated a more pronounced tubular vacuolation and interstitial edema in mice overexpressing STK25 (Figure 2C). Notably, we found that glomerulosclerosis and tubulointerstitial fibrosis were markedly aggravated in *Stk25* transgenic mice, as measured by immunofluorescence analyses of collagen IV and by Picrosirius red staining (labels both collagen I and -III) (Figure 2, D and E). Moreover, overexpression of STK25 exacerbated renal arteriolar hyalinosis, as indicated by elevated abundance of α-SMA (Figure 2F). Infiltration of cells positive for CD68 (a marker of activated macrophages) was also 2.5 ± 0.4–fold higher in the kidneys from high-fat diet–fed *Stk25* transgenic versus WT mice, demonstrating increased inflammation both in glomeruli and in the tubular area (Figure 2G). Importantly, *Stk25* transgenic kidneys displayed marked impairment in the integrity of glomerular filtration barrier (GFB), as evidenced by decreased abundance of the podocyte-specific protein nephrin and the endothelial-specific protein Pecam (3.3 ± 0.6– and 2.0 ± 0.4–fold reduction, respectively) (Figure 2, H and I).

**Figure 3:**
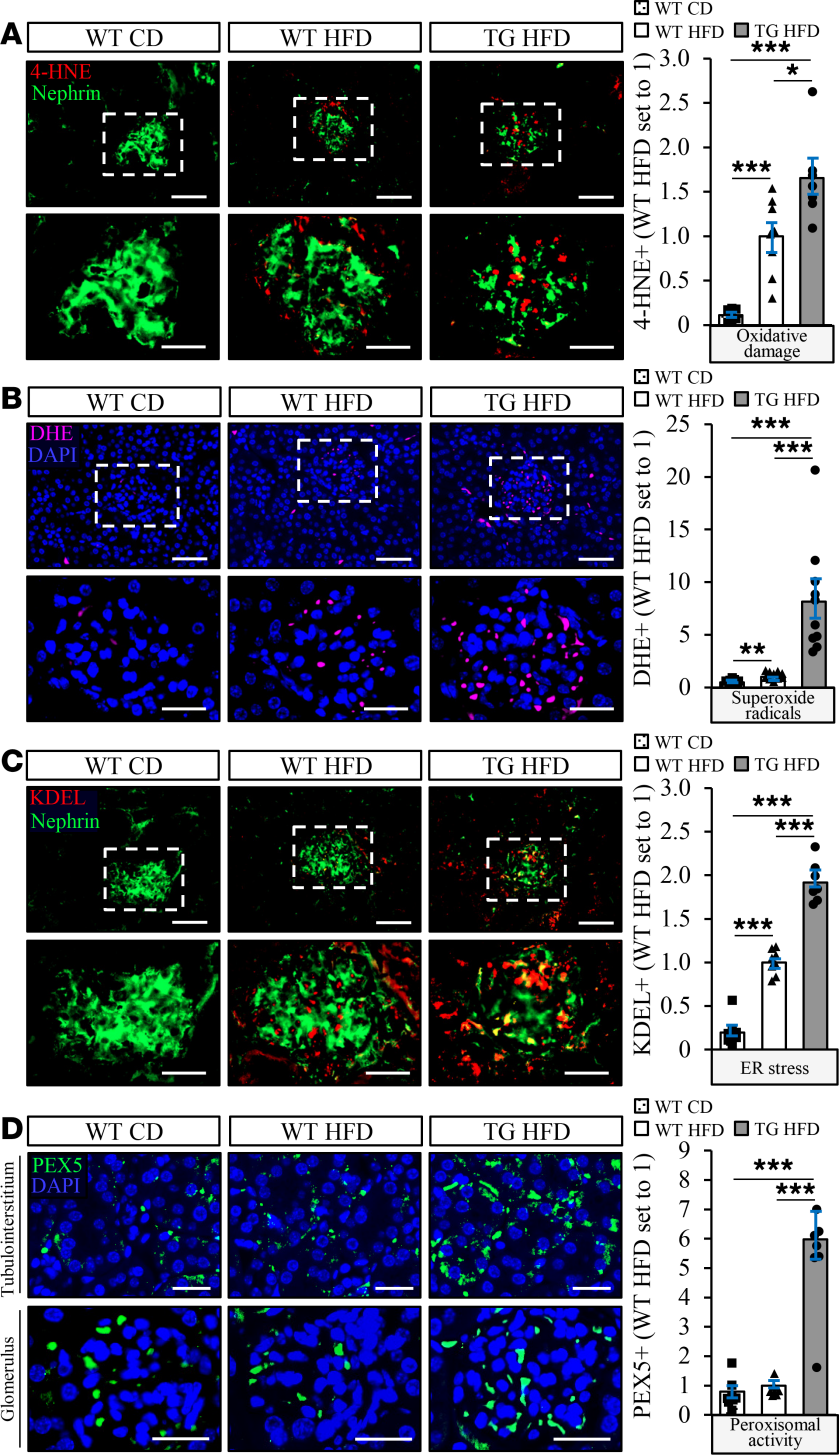
Overexpression of STK25 in high-fat diet–fed mice results in substantially increased renal oxidative and ER stress, as well as elevated peroxisomal biogenesis. (**A**–**D**) Representative kidney sections processed for immunofluorescence with anti-4-HNE (red; **A**), anti-KDEL (red; **C**), or anti-PEX5 (green; **D**) antibodies or stained with DHE (pink; **B**); nuclei stained with DAPI (blue). Quantification of the staining. In **A**–**C**, the scale bars at the top and bottom represent 50 and 25 μm, respectively; in **D**, the scale bars represent 25 μm. Data are mean ± SEM from 7–10 mice per group. CD, chow diet; HFD, high-fat diet; TG, transgenic. *P < 0.05, ***P < 0.001 by 1-way ANOVA followed by a 2-tailed Student’s *t* test

The structural changes in kidney cells characteristic of DKD are known to be preceded by exacerbated oxidative and endoplasmic reticulum (ER) stress (21). Interestingly, we found that the levels of 4-hydroxynonenal (4-HNE), an end-product of peroxidation of membrane *N*-6-polyunsaturated fatty acids and considered a reliable biomarker of oxidative damage, and the dye dihydroethidium (DHE), which detects superoxide radicals (O_2_^·^^–^), were significantly higher in kidney sections from high-fat diet–fed *Stk25* transgenic mice compared with WT controls (Figure 3, A and B). In parallel, we observed 1.9 ± 0.1–fold enhanced immunostaining for KDEL, a well-characterized ER stress indicator, in *Stk25* transgenic kidneys (Figure 3C). Notably, overexpression of STK25 also resulted in elevated renal peroxisomal activity, as evidenced by a 6.0 ± 0.7–fold increase in immunostaining for peroxisomal biogenesis marker PEX5 (Figure 3D).

No differences in body weight, or in the level of hyperglycemia or hyperinsulinemia, were detected comparing the *Stk25* transgenic and WT mice over the high-fat feeding period (Supplemental Figure 5, A–D). Furthermore, glucose tolerance and insulin sensitivity assessed at the end of the high-fat diet feeding regimen were similar between the genotypes (Supplemental Figure 5, E and F). A challenge with a high-fat diet progressively increased the levels of plasma lipids in both *Stk25* transgenic and WT mice, without any difference comparing the genotypes (Supplemental Figure 6, A–D).

### Genetic ablation of STK25 in mice halts the progression of diet-induced DKD.

**Figure 4:**
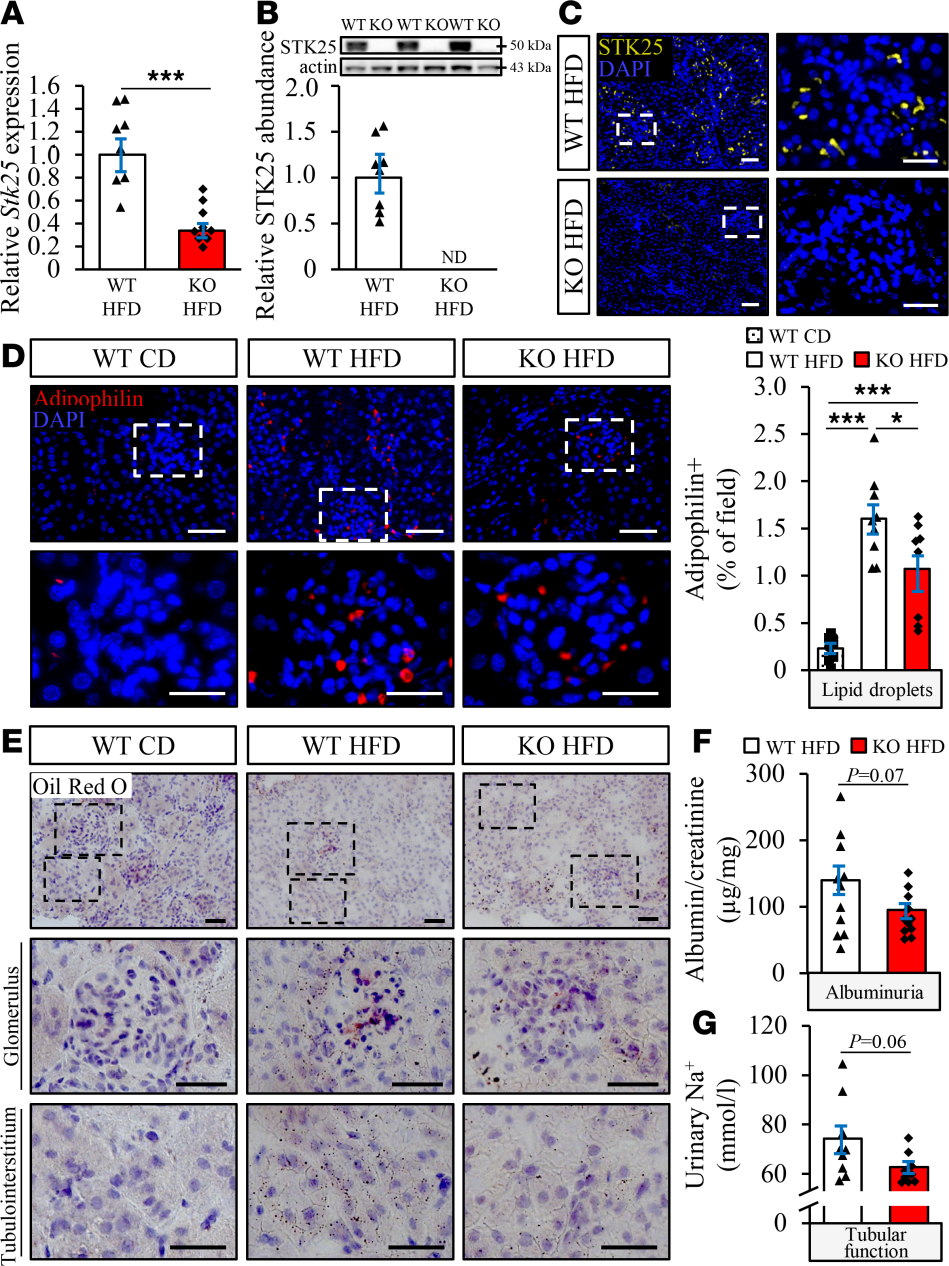
Genetic ablation of STK25 in mice prevents high-fat diet–induced renal lipid accumulation and improves kidney function. (**A** and **B**) Renal STK25 mRNA (**A**) and protein (**B**) abundance. Protein levels were analyzed by densitometry; representative Western blots are shown with pan-actin used as a loading control. (**C**) Representative kidney sections processed for immunofluorescence with anti-STK25 antibody (yellow); nuclei stained with DAPI (blue). (**D**) Representative kidney sections processed for immunofluorescence with anti-adipophilin antibody (red); nuclei stained with DAPI (blue). Quantification of the staining. (**E**) Representative kidney sections stained with Oil Red O. (**F** and **G**) Measurement of urinary ACR (**F**) and sodium levels (**G**). In **C**, the scale bars at the left and right represent 50 and 25 μm, respectively; in **D**, the scale bars at the top and bottom represent 50 and 25 μm, respectively; in **E**, the scale bars at the top and 2 bottom represent 50 and 25 μm, respectively. Data are mean ± SEM from 8–11 mice per group. CD, chow diet; HFD, high-fat diet. *P < 0.05, ***P < 0.001 by a 2-tailed Student’s *t* test in **A**, **B**, **F**, and **G**, and 1-way ANOVA followed by a 2-tailed Student’s *t* test in **D**.

To determine if reducing endogenous STK25 expression could prevent diet-induced renal lipid deposition and protect against the development of DKD, we examined the effects of *Stk25* loss of function in high-fat diet–fed mice (Figure 1A). The loss of STK25 protein expression in kidney samples from *Stk25*^–/–^ mice was confirmed by Western blot, as well as immunofluorescence analysis (Figure 4, A–C).

We found that high-fat diet–induced accumulation of lipid droplets (assessed by adipophilin immunostaining) and neutral lipids (assessed by Oil Red O staining) was reduced both in glomeruli and in the tubular area from *Stk25*^–/–^ kidneys compared with WT kidneys (Figure 4, D and E). In parallel, we detected trends of decreased albuminuria and sodium in the urine from high-fat diet–fed *Stk25*-KO mice, suggesting an improvement in glomerular permselectivity and tubular absorptive capacity (Figure 4, F and G). Notably, *Stk25*^–/–^ mice also displayed lower abundance of plasma renin and angiotensin II compared with WT littermates (Supplemental Figure 3, C and D).

**Figure 5:**
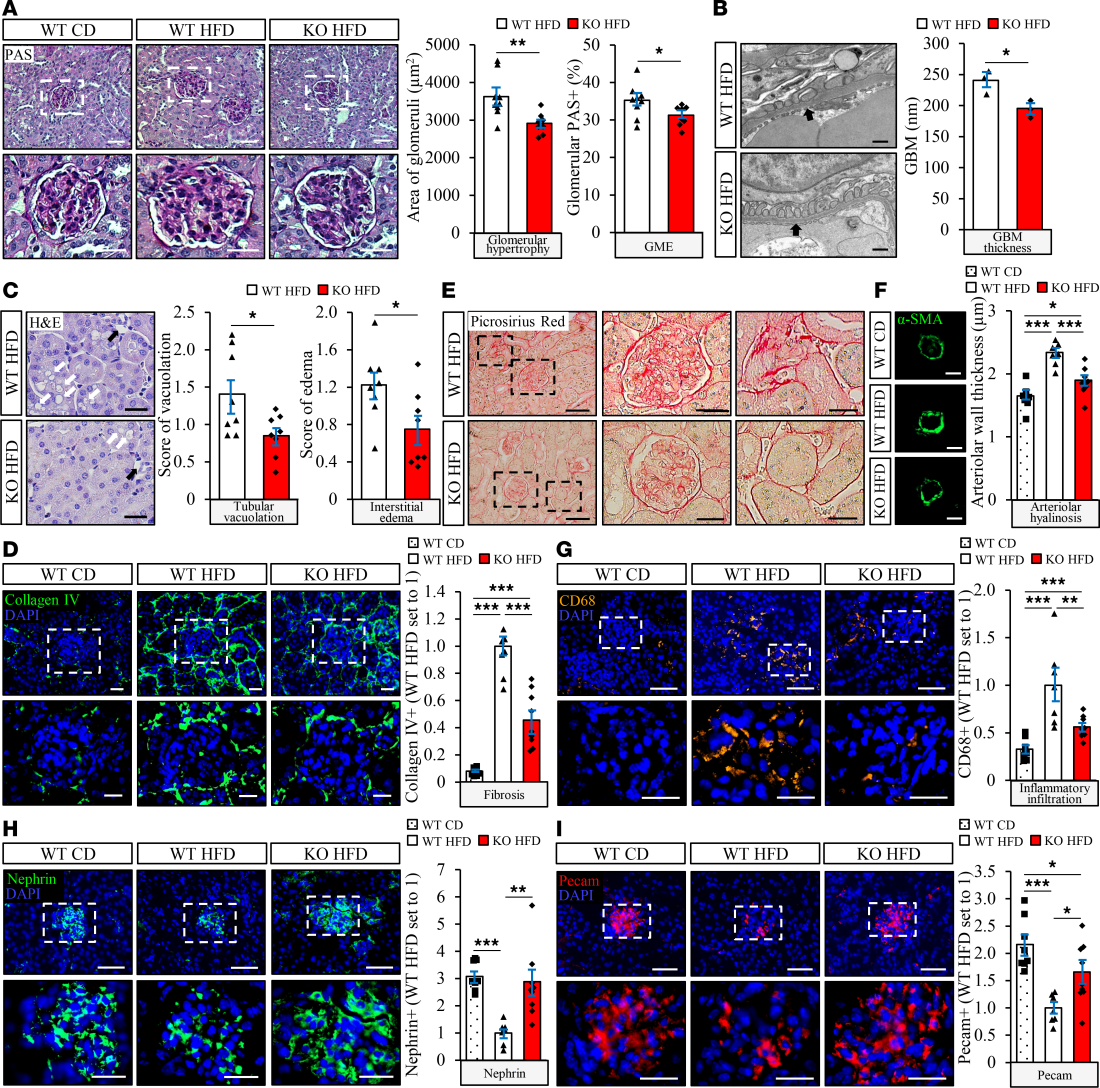
Depletion of STK25 in mice alleviates high-fat diet–induced glomerular mesangial matrix expansion, GBM thickening, tubulointerstitial injury, glomerulosclerosis and tubulointerstitial fibrosis, renal inflammation, and arteriolar hyalinosis, and it improves the integrity of GFB. (**A**) Representative kidney sections stained with PAS and quantification of glomerular hypertrophy and GME. (**B**) Representative TEM images of the kidney and measurement of the thickness of GBM (black arrows). (**C**) Representative kidney sections stained with H&E and scoring of tubular vacuolation (white arrows) and interstitial edema (black arrows). (**D**, **G**–**I**) Representative kidney sections processed for immunofluorescence with anti–collagen IV (green; **D**), anti-CD68 (yellow; **G**), anti-nephrin (green; **H**), or anti-Pecam (red; **I**) antibodies; nuclei stained with DAPI (blue). Quantification of the staining. (**E**) Representative kidney sections stained with Picrosirius red. (**F**) Representative kidney sections processed for immunofluorescence with anti–α-SMA (green) antibody and quantification of arteriolar wall thickness. In **A**, **D**, and **G**–**I**, the scale bars at the top and bottom represent 50 and 25 μm, respectively; in **B**, the scale bars represent 500 nm; in **C**, the scale bars represent 50 μm; in **E**, the scale bars at the left and middle/right represent 100 and 25 μm, respectively; in **F**, the scale bars represent 10 μm. Data are mean ± SEM from 7–9 mice per group, except in **B**, where *n* = 3 per group. CD, chow diet; HFD, high-fat diet. *P < 0.05, **P < 0.01, ***P < 0.001 by a 2-tailed Student’s *t* test in **A**–**C** and 1-way ANOVA followed by a 2-tailed Student’s *t* test in **D** and **F**–**I**.

PAS staining, as well as scanning electron microscopy and TEM analysis of kidney sections collected at the end of the high-fat diet feeding period, revealed that glomerular hypertrophy and mesangial matrix hyperplasia were significantly suppressed, whereas GBM thickness and podocyte vacuolation were decreased, in *Stk25*^–/–^ mice compared with WT littermates (Figure 5, A and B, and Supplemental Figure 4, C and D). Furthermore, depletion of STK25 lowered the scores of tubular vacuolation and interstitial edema by 1.7 ± 0.2– and 1.6 ± 0.2–fold, respectively (Figure 5C). We also detected less glomerulosclerosis and tubulointerstitial fibrosis (assessed by collagen IV immunofluorescence analysis and by Picrosirius red staining) in the kidneys from high-fat diet–fed *Stk25*-KO versus WT mice, in parallel with suppressed arteriolar hyalinosis (quantified by α-SMA immunostaining) (Figure 5, D–F). Ablation of STK25 resulted in decreased diet-induced renal inflammatory infiltration as evidenced by 1.8 ± 0.3–fold lower immunostaining for CD68 (Figure 5G). Importantly, we found 2.9 ± 0.4–fold and 1.7 ± 0.2–fold higher abundance of nephrin and Pecam, respectively, in kidney sections from high-fat diet–fed *Stk25*^–/–^ versus WT mice, suggesting a prevention of podocyte injury/loss, increased vessel density, and an improvement of GFB integrity (Figure 5, H and I).

**Figure 6:**
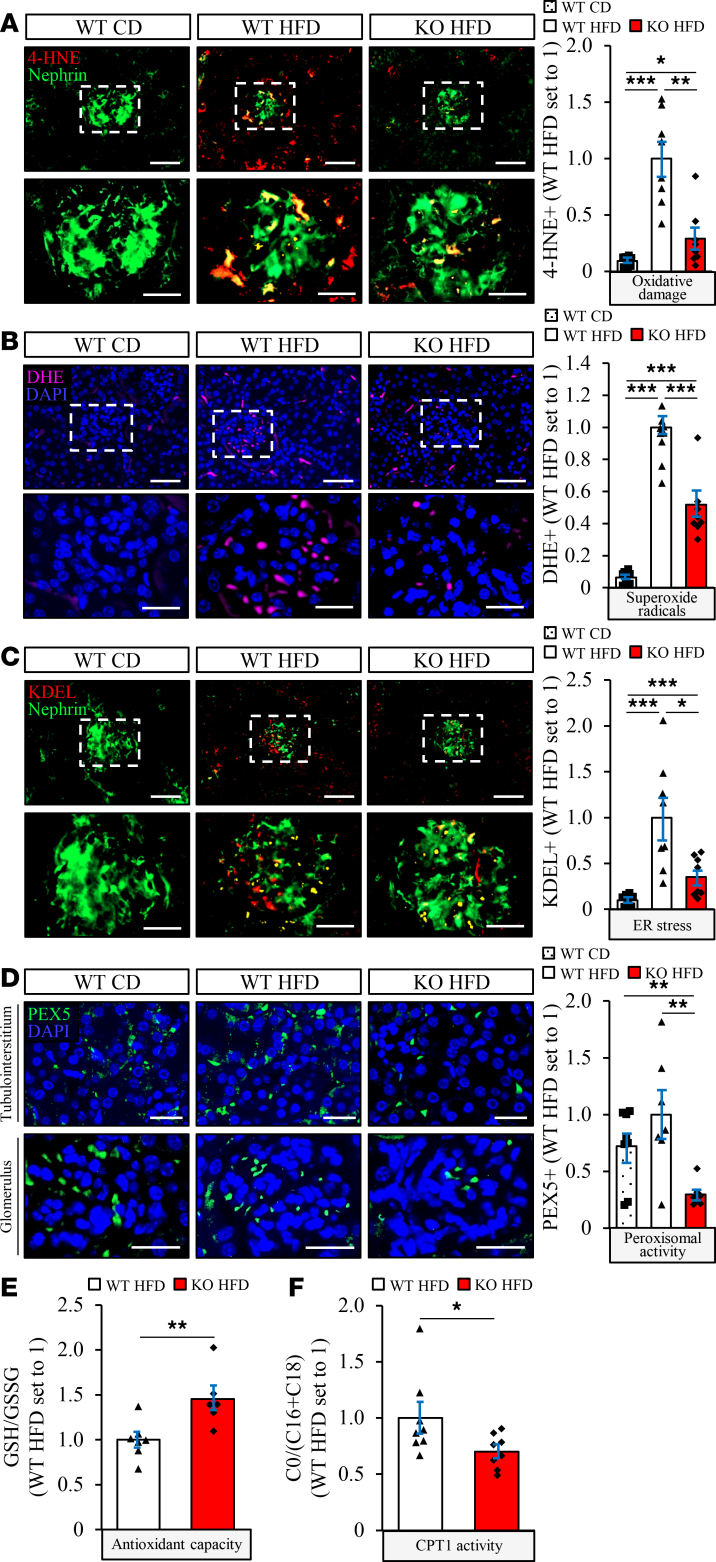
Knockdown of STK25 in high-fat diet–fed mice substantially suppresses renal oxidative and ER stress and decreases peroxisomal biogenesis. (**A**–**D**) Representative kidney sections processed for immunofluorescence with anti–4-HNE (red; **A**), anti-KDEL (red; **C**), or anti-PEX5 (green; **D**) antibodies or stained with DHE (pink; **B**); nuclei stained with DAPI (blue). Quantification of the staining. (**E**) The ratio of reduced to oxidized glutathione. (**F**) The ratio of free carnitine to the sum of palmitoylcarnitine and stearoylcarnitine. In **A**–**C**, the scale bars at the top and bottom represent 50 and 25 μm, respectively; in **D**, the scale bars represent 25 μm. Data are mean ± SEM from 6–9 mice per group. CD, chow diet; HFD, high-fat diet. *P < 0.05, **P < 0.01, ***P < 0.001 by 1-way ANOVA followed by a 2-tailed Student’s *t* test in **A**–**D** and a 2-tailed Student’s *t* test in **E** and **F**.

Reciprocally to our observations in *Stk25* transgenic mice, the oxidative stress indictors 4-HNE and DHE, as well as ER stress marker KDEL, were about 2- to 4-fold decreased both in glomeruli and in the tubular area of the kidneys from high-fat diet–fed *Stk25*^–/–^ versus WT mice (Figure 6, A–C). We also found that the ratio of reduced to oxidized glutathione (GSH/GSSG) was 1.5 ± 0.1–fold higher in kidney lysates from *Stk25*^–/–^ mice, indicating an improved antioxidant capacity (Figure 6E). Notably, 3.4 ± 0.7–fold lower PEX5 immunostaining suggested suppressed peroxisomal biogenesis in STK25-deficient kidneys (Figure 6D).

It has recently been shown that increasing fatty acid oxidation alone by genetic or pharmacological tools is sufficient to protect the kidneys from fibrotic injury (22). The rate-limiting step in β-oxidation is the transport of fatty acids into the mitochondria by carnitine palmitoyltransferase 1 (CPT1), which conjugates fatty acids with carnitine. Interestingly, we found that the ratio between free carnitine (C0) and the sum of palmitoylcarnitine and stearoylcarnitine (C16 + C18) was 1.4 ± 0.2–fold lower in kidney lysates from high-fat diet–fed *Stk25*^–/–^ mice compared with WT controls, indicating elevated CPT1 activity (Figure 6F).

The fasting blood glucose levels were similar, comparing the genotypes throughout the high-fat diet feeding experiment; however, plasma insulin and the homeostasis model assessment score of insulin resistance (HOMA-IR) were about 2-fold lower in *Stk25*^–/–^ mice than in WT littermates at the end of the feeding regimen, which was paralleled by a modest reduction in diet-induce weight gain (Supplemental Figure 7, A–D). Consistent with our previous studies (10), *Stk25*-KO mice also exhibited small but significant improvement in glucose tolerance and insulin sensitivity when assessed at the end of the high-fat feeding period (Supplemental Figure 7, E and F). Plasma lipid levels markedly increased in both *Stk25*-KO and WT mice during the period of high-fat diet feeding, with the lipid profile remaining similar between the genotypes (Supplemental Figure 6, E–H). Notably, we did not observe any differences in general behavior or clinical signs (body posture, mood, and motor activity) in high-fat diet–fed *Stk25*^–/–^ mice compared with WT controls.

### STK25 cell-autonomously controls ectopic fat accumulation in human kidney cells by regulating both lipid synthesis and oxidation.

**Figure 7:**
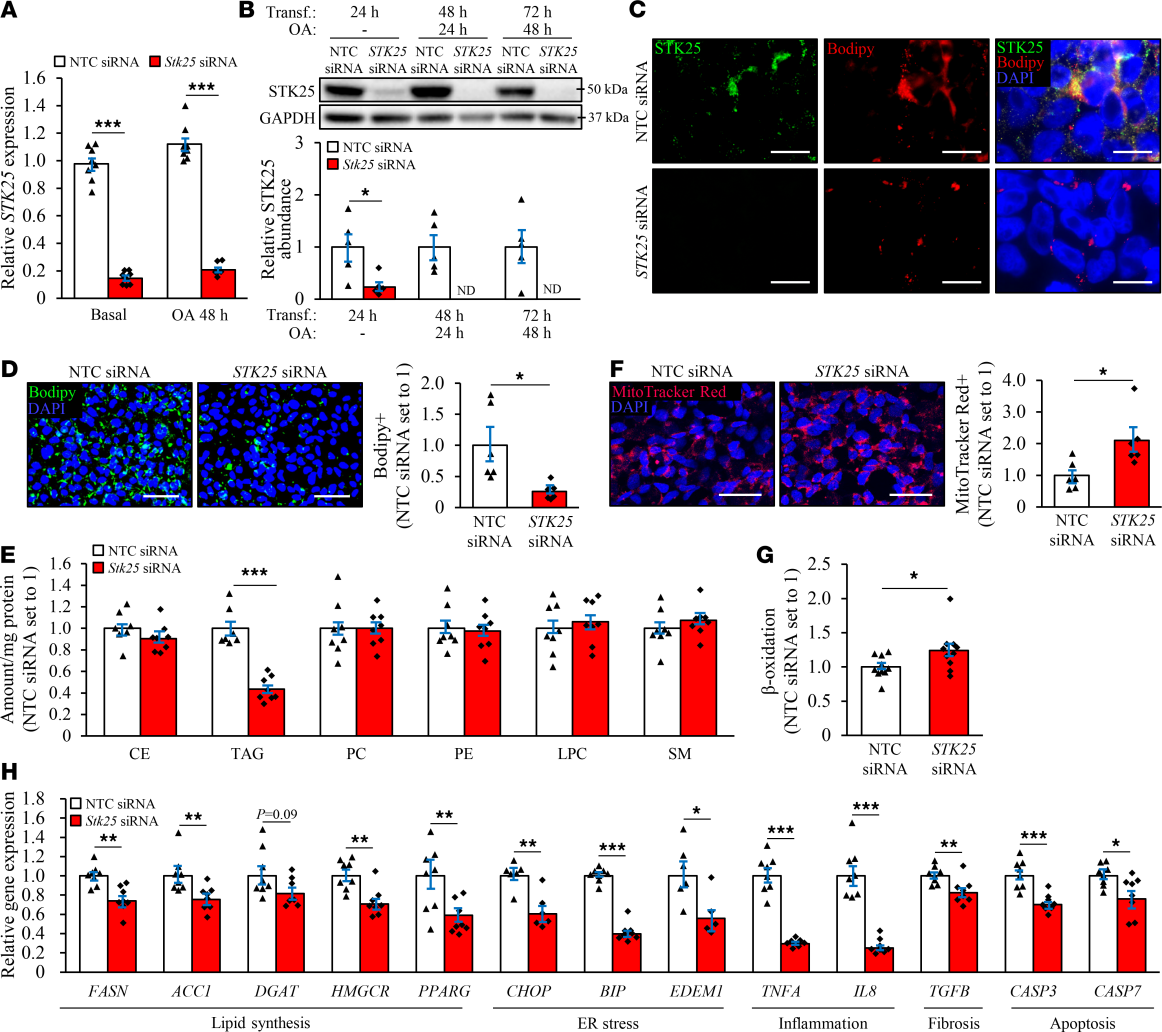
Silencing of STK25 protects HEK293 cells against ectopic lipid storage. HEK293 cells were transfected with *STK25* siRNA or NTC siRNA and challenged with oleic acid for 48 hours. (**A** and **B**) STK25 mRNA (**A**) and protein (**B**) abundance. Protein levels were analyzed by densitometry; representative Western blots are shown with GAPDH used as a loading control. (**C**) Representative images of cells processed for immunofluorescence with anti-STK25 antibody (green) and stained with Bodipy (red). Merged image shows colocalization in yellow; nuclei stained with DAPI (blue). (**D** and **F**) Representative images of cells stained with Bodipy (green; **D**) or MitoTracker Red (red, **F**); nuclei stained with DAPI (blue). Quantification of the staining. (**E**) Lipidomic analysis in cell extracts. (**G**) Oxidation of radiolabeled palmitate. (**H**) Measurement of the mRNA expression of selected markers. The gene functions are indicated at the bottom. In **C**, the scale bars represent 20 μm; in **D** and **F**, the scale bars represent 40 μm. Data are mean ± SEM from 6–10 wells per group. CE, cholesteryl ester; ND, not detected; OA, oleic acid; PC, phosphatidylcholine; PE, phosphatidylethanolamine; SM, sphingomyelin; Transf., transfection. *P < 0.05, **P < 0.01, ***P < 0.001 by a 2-tailed Student’s *t* test

To investigate the cell-specific role of STK25 in regulation of renal lipotoxicity, we transfected human embryonic kidney 293 (HEK293) cells, which are frequently used to study nephrotoxicity (23, 24), with *STK25*-specific small interfering RNA (siRNA) or with a nontargeting control (NTC) siRNA. In order to mimic conditions in high-fat diet–fed mice and high-risk individuals, we subsequently exposed the cells for 48 hours to oleic acid, known to efficiently induce steatosis in vitro. In HEK293 cells transfected with *STK25* siRNA, the target mRNA expression was repressed by approximately 80%, whereas the protein abundance of STK25 was below the Western blot detection limit (Figure 7, A and B). Similarly to our previous observations in mouse and human hepatocytes (11, 18), we found that the endogenous STK25 protein was mainly (but not solely) localized to the intracellular lipid droplets in HEK293 cells transfected with NTC siRNA (evidenced by largely colocalized immunostaining of STK25 with Bodipy 493/503 that detects neutral lipids in lipid droplets; Figure 7C). As expected, no STK25 immunostaining was detected in HEK293 cells transfected with *STK25* siRNA (Figure 7C).

The silencing of STK25 suppressed intracellular lipid deposition in HEK293 cells treated with oleic acid about 4-fold, as assessed by quantification of the Bodipy^+^ area (Figure 7D). Lipidomic analysis confirmed significantly reduced accumulation of triacylglycerol (TAG) in HEK293 cells transfected with *STK25* siRNA compared with NTC siRNA (Figure 7E). Analysis of individual TAG species further revealed that depletion of STK25 decreased the levels of all the main TAG species (Supplemental Table 1 and Supplemental Figure 8).

We next investigated the mechanisms underlying the suppression of ectopic lipid storage in HEK293 cells by STK25 knockdown. We found that the staining with MitoTracker Red, a fluorescent dye that specifically accumulates within respiring mitochondria, was significantly augmented in oleate-treated HEK293 cells transfected with *STK25* siRNA versus NTC siRNA (Figure 7F). Consistently, silencing of STK25 resulted in about a 25% increase in β-oxidation rate measured by quantification of [^3^H]-labeled water as the product of (9,10-^3^H[N]) palmitic acid oxidation (Figure 7G). Notably, the mRNA levels of FASN and ACC1 controlling de novo fatty acid synthesis, and DGAT and HMGCR catalyzing the commitment steps in TAG and cholesterol biosynthesis, respectively, were lower in HEK293 cells transfected with *STK25* siRNA, along with reduced expression of lipogenic transcription factor PPARγ (Figure 7H).

Interestingly, we also detected repressed intracellular lipid storage, increased mitochondrial β-oxidation, and suppressed lipogenic gene expression in STK25-deficient HEK293 cells cultured without oleate supplementation; however, the effect of STK25 silencing was less pronounced in cells maintained under basal conditions (Supplemental Figure 9, A–D).

Consistent with lower fat storage in STK25-deficient HEK293 cells, we found substantially reduced intracellular lipid deposition in oleate-treated human proximal tubular (HK-2) cells, mesangial cells, and podocytes transfected with *STK25* siRNA versus NTC siRNA (Supplemental Figure 10, A, B, E, F, I, J).

### Silencing of STK25 protects human kidney cells against oxidative and ER stress and activates autophagy.

**Figure 8:**
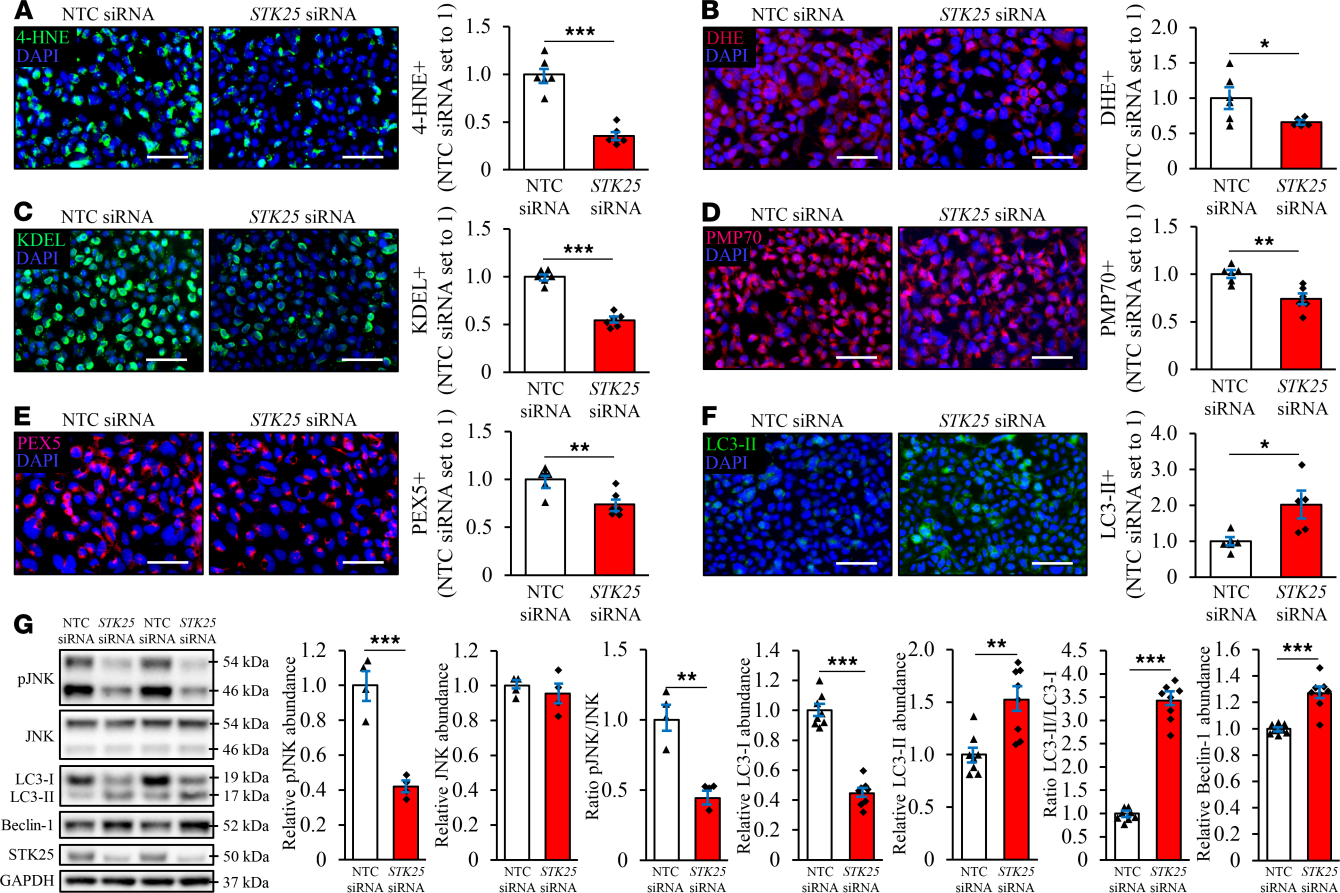
Silencing of STK25 protects HEK293 cells against oxidative and ER stress, and it activates autophagy. HEK293 cells were transfected with *STK25* siRNA or NTC siRNA and challenged with oleic acid for 48 hours. (**A**–**F**) Representative images of cells processed for immunofluorescence with anti–4-HNE (green; **A**), anti-KDEL (green; **C**), anti-PMP70 (red; **D**), anti-PEX5 (pink; **E**), or anti-LC3 (green, **F**) antibodies or stained with DHE (red; **B**); nuclei stained with DAPI (blue). Quantification of the staining. (**G**) Cell lysates were analyzed by Western blot using antibodies specific for phospho-JNK (Thr^183^/Tyr^185^), JNK, LC3, Beclin-1, and STK25. Protein levels were analyzed by densitometry; representative Western blots are shown with GAPDH used as a loading control. In **A**–**F**, the scale bars represent 40 μm. Data are mean ± SEM from 4–8 wells per group. *P < 0.05, **P < 0.01, ***P < 0.001 by a 2-tailed Student’s *t* test

Lipid accumulation is known to display direct toxic effect on renal cells by initiating oxidative and ER stress, leading to a vicious cycle of inflammation, fibrosis, and apoptosis (21, 25). Indeed, we found that, in parallel with reduced lipid storage, silencing of STK25 protected HEK293 cells challenged with oleic acid against oxidative damage, as evidenced by suppressed lipid peroxidation (2.8 ± 0.2–fold lower 4-HNE^+^ area and 1.5 ± 0.2–fold lower TBARS concentration; Figure 8A and Supplemental Figure 11) and decreased superoxide anion levels (1.5 ± 0.2–fold lower DHE^+^ area; Figure 8B). We also detected 1.8 ± 0.1–fold reduction in immunostaining for ER stress marker KDEL in STK25-deficient HEK293 cells treated with oleic acid (Figure 8C). Furthermore, the mRNA abundance of the major indicators of ER stress CHOP (also known as DDIT3), BIP (also known as HSPA5 or GRP78), and EDEM1 was significantly lower in HEK293 cells transfected with *STK25* siRNA compared with NTC siRNA, along with reduced expression of proinflammatory cytokines TNF-α and IL-8, as well as profibrotic cytokine TGF-β (Figure 7H). Consistently, the mRNA expression of proapoptotic markers caspase-3 and -7 (CASP3 and CASP7) was decreased in STK25-deficient HEK293 cells (Figure 7H), and the level of activated c-Jun–N-terminal kinase (phospho-JNK Thr^183^/Tyr^185^), an important downstream mediator of lipoapoptosis, was markedly lower (Figure 8G). Similar to our findings in the kidneys from *Stk25*^–/–^ mice, the silencing of STK25 in oleate-treated HEK293 cells also suppressed the peroxisomal activity, as evidenced by 1.4 ± 0.1–fold lower immunostaining for peroxisomal membrane protein PMP70 and peroxisomal biogenesis marker PEX5 (Figure 8, D and E). Notably, reduced oxidative and ER stress, as well as decreased peroxisomal function, were observed even in STK25-deficient HEK293 cells cultured without oleic acid supplementation in the culture media (Supplemental Figure 9, E–I).

Consistent with these findings in HEK293 cells, we detected significantly suppressed oxidative and ER stress assessed by immunostaining for 4-HNE and KDEL, respectively, in oleate-treated HK-2 cells, mesangial cells, and podocytes transfected with *STK25* siRNA versus NTC siRNA (Supplemental Figure 10, C, D, G, H, K, L).

To elucidate whether the increased STK25 abundance in human kidney cells leads to a reciprocal effect compared with STK25 knockdown (i.e., aggravated oxidative stress and increased peroxisomal activity), we transfected HEK293 cells with human *STK25* expression plasmid or empty control plasmid. Cells transfected with *STK25* expression plasmid displayed about 5- to 7-fold higher STK25 protein abundance (Supplemental Figure 12A). Indeed, we found markedly elevated oxidative damage (assessed by 4-HNE^+^ and DHE^+^ area) in STK25-overexpressing HEK293 cells, both with and without oleate challenge, in parallel with enhanced peroxisomal activity (assessed by PMP70^+^ and PEX5^+^ area) (Supplemental Figure 12, B–E).

Recent studies have provided indirect and direct evidence that downregulation of autophagy is involved in the pathogenesis of DKD, including both glomerular and tubulointerstitial lesions (26, 27). To evaluate the possible role of autophagy in the protective effects observed in STK25-deficient kidney cells, we next compared the protein abundance of autophagic markers in oleate-treated HEK293 cells transfected with *STK25* siRNA versus NTC siRNA. Western blot analysis revealed that the silencing of STK25 increased the conversion of LC3-I to LC3-II about 3.5-fold, which is considered a key marker of enhanced autophagic flux, and significantly elevated the protein levels of autophagy inducer Beclin-1 (Figure 8G). Consistently, there were more LC3-II^+^ puncta in HEK293 cells transfected with *STK25* siRNA compared with NTC siRNA (Figure 8F). Again, the activation of autophagy in STK25-deficient HEK293 cells was detected independently of oleate supplementation in the culture media (Supplemental Figure 9, J and K).

## Discussion

**Figure 9:**
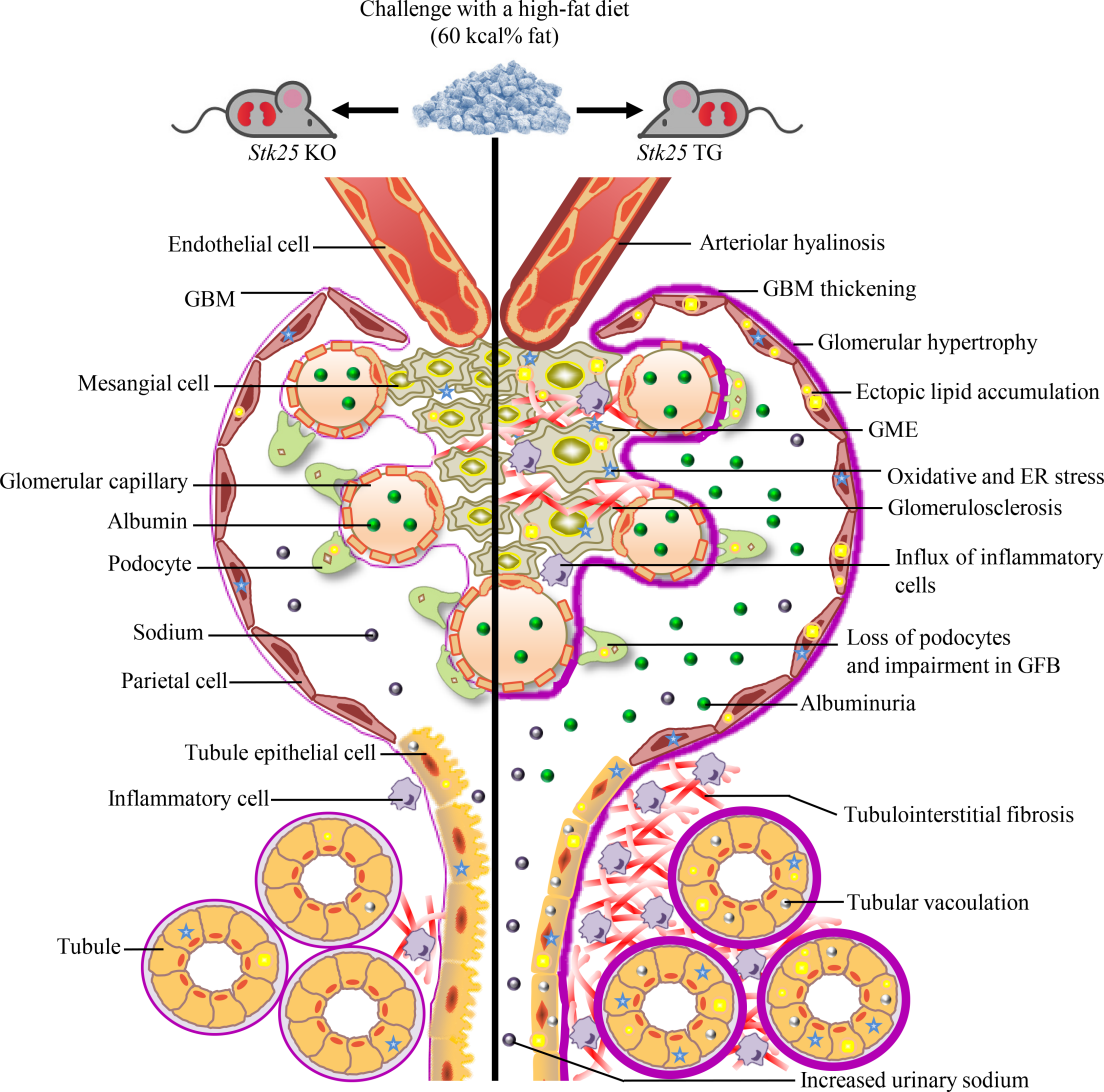
Schematic illustration of the impact of STK25 signaling on susceptibility to lipotoxicity-mediated progression of DKD. Overexpression of STK25 in mice challenged with a high-fat diet triggers DKD-associated pathologies; reciprocally, genetic ablation of STK25 in high-fat diet–fed mice is sufficient to protect against glomerular and tubulointerstitial structural and functional changes characteristic of DKD. TG, transgenic.

This study unravels a role of protein kinase STK25 in the development and progression of DKD. We found that overexpression of STK25 in mice challenged with a high-fat diet triggered DKD-associated pathologies, including exacerbated glomerular mesangial matrix expansion, GBM thickening, and impairment in the integrity of GFB, vacuolar degeneration of tubular cells and interstitial edema, worsened glomerulosclerosis and tubulointerstitial fibrosis, aggravated renal arteriolar hyalinosis, and elevated albuminuria (Figure 9). Reciprocally, we observed that the genetic ablation of STK25 was sufficient to attenuate glomerular and tubulointerstitial injury and preserve kidney function in a mouse model with diet-induced DKD (Figure 9). A marked impact of altered STK25 signaling on structural and functional damage of glomerular and tubular cells is consistent with the high expression levels of endogenous STK25 in both these compartments.

Interestingly, we found that exacerbated diet-induced kidney injury in *Stk25* transgenic mice was associated with excessive lipid storage both in glomeruli and in the tubular area compared with WT controls. Reciprocally, the renoprotective effects observed in high-fat diet–fed *Stk25*^–/–^ mice were accompanied by attenuated lipid deposition in the kidney. To date, the molecular mechanisms controlling renal lipid accumulation remain elusive; however, renal steatosis has been shown to develop when lipogenesis in the kidney is enhanced or fatty acid oxidation is compromised (22, 28–32). Notably, kidneys preferentially oxidize fatty acids as energy sources and are not a major contributor to circulating glucose catabolism (22). Here, we found that silencing of STK25 in cultured human kidney cells also suppressed intracellular lipid storage, and this was paralleled by reduced expression of key enzymes catalyzing the commitment steps in lipid synthesis pathway and, reciprocally, substantially enhanced mitochondrial β-oxidation rate. Taken together, these results suggest that inhibition of STK25 signaling can ameliorate diet-induced renal steatosis by directly reprograming cell metabolism with a decrease in lipogenesis and an increase in mitochondrial biogenesis. Consistent with our observations in kidney cells, depletion of STK25 in mouse and human hepatocytes was previously demonstrated to suppress lipid droplet anabolism through reduced TAG synthesis and enhance lipid droplet catabolism through elevated β-oxidation and very low–density lipoprotein–TAG (VLDL-TAG) secretion (11, 14, 18). Importantly, the fasting plasma concentrations of TAG and cholesterol are similar, comparing high-fat diet–fed *Stk25* transgenic and -KO mice with their WT littermates (this study; refs. 10, 33), suggesting that differences in circulating lipids did not contribute to the alterations in renal or hepatic fat storage.

Recent evidence accumulated in humans and experimental animals suggests a key role of renal lipids in structural and functional injury of both glomerular and tubular cells to propose the development of obesity-related DKD (34). Although the precise mechanisms linking renal lipotoxicity to the initiation and progression of DKD are not fully understood, the aggravated oxidative and ER stress triggered by excessive lipid storage has been shown to display direct toxic effects on kidney cells and induce local inflammation and fibrosis, ultimately leading to renal failure (21, 35–39). Notably, apart from exaggerated kidney damage with excessive inflammatory infiltration and fibrosis, we also found substantially increased oxidative and ER stress both in glomeruli and in the tubular area of high-fat diet–fed *Stk25* transgenic mice compared with WT controls. In contrary, protection against DKD-associated pathologies in *Stk25*-KO mice was paralleled by alleviated renal oxidative and ER stress. Consistent with our observations in *Stk25*^–/–^ mice, silencing of STK25 in cultured human kidney cells also reduced oxidative damage and suppressed the abundance of ER stress indicators and proapoptotic markers. Hence, our data suggest that inhibition of STK25 activity directly attenuates the initiation and progression of DKD by ameliorating lipotoxicity-induced oxidative and ER stress in kidney cells.

Similarly to renal lipotoxicity, obesity-related fat accumulation within the liver and pancreas (i.e., nonalcoholic fatty liver disease [NAFLD] and nonalcoholic fatty pancreas disease [NAFPD], respectively) has emerged as a key causative factor in local inflammation and fibrogenic process, leading to cell damage and apoptosis (40, 41). Intramyocellular lipid storage is also known to impair insulin action in skeletal muscle, contributing to the pathogenesis of type 2 diabetes (42), whereas lipid deposition in the vascular intima results in progression to complex atherosclerotic lesions, predisposing the patients to cardiovascular diseases, such as myocardial infarction and stroke (43). Importantly, in addition to the direct impact of STK25 on diet-induced renal steatosis and kidney injury described in this study, our previous investigations have revealed that STK25 also critically controls lipid accumulation, metainflammation, and nutritional fibrosis in liver, pancreas, skeletal muscle, and aorta in the context of obesity (10–17). Thus, STK25 emerges as a key regulator governing susceptibility to lipotoxicity, and an inhibition of STK25 activity may prevent various complex metabolic diseases that are associated with ectopic lipid deposition in peripheral tissues, including kidney. Notably, we have shown earlier that high-fat diet–fed *Stk25*^–/–^ mice also display improved mitochondrial function and suppressed diet-induced cell hypertrophy, inflammatory infiltration, and extracellular matrix deposition in brown and white adipose depots (44), suggesting that repression of STK25 signaling may even contribute to establishing a healthier adipose tissue.

Interestingly, we found that peroxisomal biogenesis was markedly suppressed in the kidneys from *Stk25*^–/–^ mice and in STK25-deficient human kidney cells, and the reciprocal phenotype was seen when STK25 was overexpressed. Impaired renal peroxisomal function has been shown to increase ROS production in several studies (45, 46); however, here we found that lower peroxisomal biogenesis in STK25-deficient kidney cells clearly correlated with substantially reduced oxidative damage. Notably, our recent studies using global proteomic analysis also revealed that a number of proteins involved in peroxisomal function, including mediators of peroxisomal fatty acid transport, metabolism, and β-oxidation, are coordinately suppressed in the livers from high-fat diet–fed *Stk25*^–/–^ mice, which is paralleled by alleviated ROS production and protection against hepatic inflammation, fibrosis, and cell damage (47). Thus, our data suggest that suppressed peroxisomal biogenesis — at least when paralleled with an increase in mitochondrial activity, as seen in STK25-deficient kidney and liver cells (11, 18) — may result in reduced rather than increased oxidative stress.

The results of this study also reveal that autophagy was significantly induced by inhibition of STK25 signaling in human kidney cells, which we observed both in basal culture conditions and when cells were challenged by oleic acid. Autophagy has been demonstrated to play an essential role in protecting the renal cells against injury under diabetic conditions through removal of protein aggregates and damaged organelles, as well as by promoting cell survival; the activation of autophagy has recently been suggested as a potential new therapeutic strategy for DKD (26, 48–51). Autophagy is thought to be especially important for maintaining the homeostasis of postmitotic cells, such as podocytes, which have only limited capacity for regeneration and display a high level of basal autophagy (27, 52–54). However, the autophagic process is a very complicated pathway, and persistent overactivation of autophagy can also exaggerate renal injury (55–57). It is not currently known whether the silencing of STK25 also enhances the autophagy flux in extrarenal tissues prone to diabetic damage.

The whole-body overexpression and depletion of STK25 in transgenic and KO mice, respectively, do not allow us to conclude whether the impact of STK25 on the kidney damage is direct or secondary to the action of this kinase in extrarenal tissues, which is a limitation of the models used. Notably, although the fasting blood glucose levels remained similar in *Stk25*^–/–^ and WT mice in this study, *Stk25*-KO mice exhibited suppressed hyperinsulinemia and an increase in whole-body glucose tolerance and insulin sensitivity at the end of the high-fat feeding period. Thus, it cannot be excluded that these alterations in glucose and insulin homeostasis contributed to the improvement in renal function observed in *Stk25*^–/–^ mice. In contrast, we detected no differences in circulating glucose or insulin concentrations, or glucose tolerance or insulin sensitivity, in high-fat diet–fed *Stk25* transgenic versus WT mice, suggesting that overexpression of STK25 aggravated the development of diet-induced DKD in mice independent of changes in systemic glucose and insulin homeostasis. Importantly, in vitro experiments performed in different human kidney cell lines in this study clearly support the direct renoprotective role of STK25 antagonism.

In contrast to our observations in this study, we previously found that transgenic mice overexpressing STK25, when challenged with a high-fat diet, are characterized by elevated fasting plasma insulin, and impaired systemic glucose tolerance and insulin sensitivity, compared with WT controls (33). Furthermore, in addition to improved glucose tolerance and insulin sensitivity, as well as lower plasma insulin concentration detected in *Stk25-*KO mice in this study, we previously also observed reduced hyperglycemia in high-fat diet–fed *Stk25*^–/–^ versus WT mice (10). Importantly, even though the mice were challenged with a pelleted high-fat diet in all these studies, the composition of the obesogenic diets was not identical (a diet with 60 kcal% derived from fat was used in the current study, compared with a diet with 45 kcal% derived from fat applied in our previous studies; refs. 10, 33). It is therefore likely that the differences in glucose and insulin homeostasis observed comparing these cohorts of mice are explained by the differences in the dietary regimens applied.

STK25 belongs to the GCKIII subfamily of sterile 20 kinases together with the 2 closely related proteins MST3 and MST4 (9). Notably, we found that depletion of STK25 in the kidneys of KO mice did not result in any compensatory increase in the expression levels of MST3 or MST4 (Supplemental Figure 13, A and B). Even though the localization of STK25 and MST3/4 in the kidney overlaps (58), and these proteins share high-sequence homology (9), the results of this study clearly show that the presence of MST3 and MST4 in kidney cells cannot compensate for the loss of STK25 in regulation of renal lipid homeostasis and metabolic stress. Thus, our study establishes a unique role of STK25 in DKD.

The current treatment paradigms for DKD, mainly involving glucose lowering and antihypertensive therapies, are not sufficiently effective, and DKD still poses a major risk factor for the development of ESRD (59). Hence, new pharmacological approaches are urgently needed to counteract progressive renal decline in patients with diabetes. Importantly, the mechanisms underlying the initiation and progression of DKD have not been fully delineated, which hampers the development of new anti-DKD therapies. Here, we provide compelling evidence for essential and multiple roles of protein kinase STK25 in DKD in the context of obesity and show that antagonizing the STK25 signaling can ameliorate diet-induced renal lipotoxicity and, as a consequence, preserve the glomerular and tubular structural and functional integrity. Whereas the phenotypes across the studied mouse strains and kidney cell lines are consistent with a critical role for STK25 in DKD, we have not yet fully resolved the mechanism of action of STK25 in the kidney tissue or its potential substrates. Future studies are also needed to address the potential therapeutic relevance of pharmacological STK25 antagonism as a strategy to dampen or abrogate the development of DKD in humans.

## Methods

### Animal experiments and metabolic measurements.

*Stk25* transgenic and -KO mice were generated and genotyped as previously described (33, 60). In all experiments, *Stk25* transgenic and -KO mice were compared with their corresponding WT littermates only because the genetic background of these lines differs (C57BL6/N for *Stk25* transgenic mice and C57BL6/J for *Stk25*^–/–^ mice). The male transgenic and KO mice and their corresponding WT littermates were weaned at 3 weeks of age and housed 3–5 mice per cage in a temperature-controlled (21°C) facility with a 12-hour light-dark cycle and free access to chow and water. From the age of 6 weeks, the mice were fed a pelleted high-fat diet (60 kcal% fat; D12492; Research Diets) for 20 weeks. Age-matched chow-fed mice served as lean controls. Body weight, fasting blood glucose and plasma insulin levels, i.p. glucose tolerance tests (GTT), and i.p. insulin tolerance tests (ITT) were assessed as previously described (15). Urine was collected, and albumin and creatinine levels were determined using the Mouse Albumin ELISA Kit and the Creatinine Assay Kit (Abcam), respectively; urinary sodium levels were measured using the Sodium Assay Kit (Abcam). At the age of 26 weeks, the mice were euthanized after withholding food for 4 hours. Blood was collected by heart puncture. Plasma levels of renin and angiotensin II were determined using murine ELISA kits (Abcam, ab138875; MilliporeSigma, RAB0010). Plasma TAG, cholesterol, and free fatty acid levels were measured using the Triglyceride Colorimetric Assay Kit (Cayman Chemical), Cholesterol Quantitation Kit (MilliporeSigma), and Free Fatty Acid Quantitation Kit (MilliporeSigma), respectively. Plasma lipoproteins were separated in pooled samples from each group by size-exclusion chromatography, and cholesterol was quantified with a system of detection online as described (61). Kidney samples were collected for histologic analysis or snap-frozen in liquid nitrogen and stored at –80°C for analysis of gene and protein expression and biochemical assays.

### Histological analysis of kidneys sections.

Kidney samples from mice were fixed with 4% (vol/vol) phosphate buffered formaldehyde (Histolab Products), embedded in paraffin, sectioned, and stained with PAS (MilliporeSigma), Picrosirius red (Histolab Products), or H&E (Histolab Products). Sections stained with H&E were used to determine tubular injury in 20 randomly selected microscopic fields (20×) per mouse as previously described (20, 62). Kidney samples were also embedded in optimal cutting temperature mounting medium (Histolab Products) and frozen in liquid nitrogen, followed by cryosectioning. Cryosections were stained with Oil Red O (MilliporeSigma) for neutral lipids or DHE (Invitrogen) to assess oxidative stress as previously described (12). For immunofluorescence analysis, kidney sections were incubated with primary antibodies, followed by incubation with fluorescent-dye conjugated secondary antibodies (see Supplemental Table 2 for antibody information). The stained area was quantified in 6–8 randomly selected microscopic fields (20×) per mouse using the ImageJ software (1.47v; NIH). Electron microscopy analysis of kidney tissue was performed by scanning electron microscopy and TEM (Zeiss Gemini II 450 with Atlas software, Zeiss; Thermo Fisher Scientific Talos L120 C with 4x4k Ceta camera, Thermo Fisher Scientific). Prior to electron microscopy analysis, the kidney cortex pieces from mice perfused with modified Karnovsky fixative (2.5% glutaraldehyde, 2% formaldehyde, 0.02% sodium azide in 0.05 mol/L sodium cacodylate buffer) were subjected to microwave-assisted processing (Leica EM AMW; Leica Microsystems) in previously described reagents (63).

### Biochemical analysis of kidney samples.

Phase extraction of metabolites from the frozen kidney samples of mice was performed as previously described (64). The AbsoluteID p180 Kit (Biocrates Life Sciences) was used to determine 186 metabolites. The samples were measured on mass spectrometer QTRAP 4500 (Sciex), in combination with a high-performance liquid chromatography (Agilent Technologies). The concentrations of the metabolites were calculated automatically by the MetIDQ software (Biocrates Life Sciences). The levels of reduced glutathione and oxidized glutathione were quantified with a luminescence-based glutathione assay (GSH-Glo; Promega). See Supplemental Table 3 for the summary of all significantly altered metabolites in the kidneys from *Stk25*^–/–^ versus WT mice.

### Culture, transient transfections, and assessments of human kidney cells.

HEK293 cells (American Type Culture Collection) and human mesangial cells (Lonza) were maintained in DMEM GlutaMAX (high glucose; Thermo Fisher Scientific) and RPMI 1640 medium (Thermo Fisher Scientific), respectively, supplemented with 10% (vol/vol) FBS and 1% (vol/vol) penicillin/streptomycin (Thermo Fisher Scientific). HK-2 cells (human immortalized proximal tubule epithelial cell line; American Type Culture Collection) were maintained in keratinocyte serum-free medium, supplemented with 109 μmol/L bovine pituitary extract and 4 μmol/L human recombinant epidermal growth factor (Thermo Fisher Scientific). Human immortalized podocytes (a gift from Professor Moin Saleem, University of Bristol, Bristol, United Kingdom) were grown and differentiated as previously described (65). Cells were demonstrated to be free of mycoplasma infection by use of the MycoAlert Mycoplasma Detection Kit (Lonza). For RNA interference, cells were transfected with human *STK25* siRNA (s20570; Ambion) or scrambled siRNA (SIC001; MilliporeSigma) using Lipofectamine RNAiMax (Thermo Fisher Scientific). For transient overexpression, cells were transfected with p*FLAG*-*STK25* (GeneCopoeia) or an empty control plasmid using Lipofectamine 2000 (Thermo Fisher Scientific). In most experiments, cells were exposed to 50 μmol/L oleic acid (MilliporeSigma) for 48 hours before assessments.

Cells were stained with Bodipy 493/503 (Invitrogen) for neutral lipids or MitoTracker Red (Thermo Fisher Scientific) for active mitochondria, as previously described (58). For DHE staining, cells were incubated with 5 μmol/L DHE (Invitrogen) in PBS containing 1% (weight/vol) BSA at 37°C for 5 minutes. Cells were also processed for immunofluorescence with anti-STK25, anti–4-HNE, anti-KDEL, anti-PMP70, anti-PEX5, or anti-LC3 antibodies (see Supplemental Table 2 for antibody information). The labeled area was quantified in 6–10 randomly selected microscopic fields (10×, 20×, or 40×) per well using the ImageJ software. To measure β-oxidation, cells were incubated in the presence of (9,10-^3^H[N]) palmitic acid, and (^3^H)-labeled water was quantified as the product of free fatty acid oxidation (18). Thiobarbituric acid–reactive substance (TBARS) levels were measured in cell extract using the TBARS Assay Kit (MilliporeSigma). Lipids were also extracted using the BUME method (66) and quantified using direct infusion on a QTRAP 5500 mass spectrometer (Sciex) equipped with a robotic nanoflow ion source, the TriVersa NanoMate (Advion BioSciences).

### Quantitative PCR.

RNA was isolated from mouse kidneys and HEK293 cells with the EZNA Total RNA Kit (Omega Bio-Tek) according to the manufacturer’s recommendations. cDNA was synthesized using the High-Capacity Complementary DNA Reverse Transcription Kit (Applied Biosystems). Relative quantification was performed with the CFX Connect Real-Time System (Bio-Rad). Relative quantities of target transcripts were calculated after normalization of the data to the endogenous control, 18S ribosomal RNA (Applied Biosystems).

### Western blot.

Western blot was performed as previously described (33) (see Supplemental Table 2 for antibody information).

### Statistics.

Statistical significance between the groups was evaluated using the 2-tailed Student’s *t* test and, among more than 2 groups, by 1-way ANOVA followed by a 2-tailed Student’s *t* test for post hoc analysis. The Shapiro-Wilk’s and the Levene’s tests were applied to confirm the normality of distribution of residuals and the homogeneity of variances, respectively. Differences were considered statistically significant at *P* < 0.05. All statistical analyses were performed using SPSS statistics (v24; IBM Corporation).

### Study approval.

All animal experiments were performed after prior approval from the local ethics committee. The mice received humane care according to the NIH recommendations outlined in the *Guide for the Care and Use of Laboratory Animals* (National Academies Press, 2011).

## Author contributions

EC, MC, and LG generated the bulk of the results and reviewed and edited the manuscript. NMK and AN contributed to the research data and reviewed and edited the manuscript. MS performed the lipidomics analysis and reviewed and edited the manuscript. RP and US performed the metabolic analysis and reviewed and edited the manuscript. MP and PP examined TAG and cholesterol distribution across the lipoprotein fractions and reviewed and edited the manuscript. HUM, JN, and JB provided advice and expertise, contributed to the discussion, and reviewed and edited the manuscript. BWH provided advice and reagents and reviewed and edited the manuscript. MM directed the project, designed the study, interpreted the data, and wrote, reviewed, and edited the manuscript. MM is the guarantor of this work and, as such, had full access to all the data in the study and takes responsibility for the integrity of the data and the accuracy of the data analysis.

## Supplementary Material

Supplemental data

## Version 1 11/10/2020

In-Press Preview

## Version 2 12/17/2020

Electronic publication
