# Supplementary material for: Depletion of protein kinase STK25 ameliorates renal lipotoxicity and protects against diabetic kidney disease
Source: JCI Insight. 2020 Dec 17;5(24):e140483. doi: 10.1172/jci.insight.140483 (PMC7819747; doi:10.1172/jci.insight.140483)
Supplement: Supplemental data [file jciinsight-5-140483-s105.pdf]

Supplementary information

Depletion of Protein Kinase STK25 Ameliorates Renal Lipotoxicity and Protects Against Diabetic Kidney Disease

Cansby et al.

Figure S1

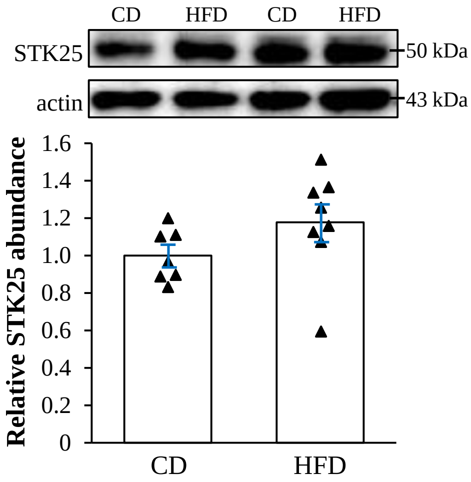

Fig. S1. The protein levels of STK25 in the kidney are not affected by the diet. STK25 protein abundance in the kidneys from wild-type mice fed a chow or a high-fat diet. Protein levels were analyzed by densitometry; representative Western blots are shown with pan-actin used as a loading control. Data are mean ± SEM from 7-8 mice per group. CD, chow diet; HFD, high-fat diet.

Figure S2

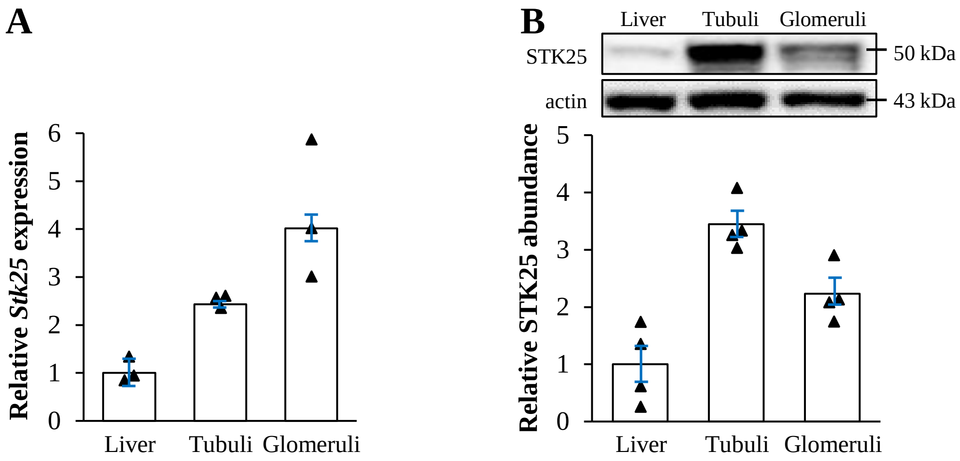

Fig. S2. Endogenous STK25 protein is detected in both glomerular and renal tubular cells. (A-B) STK25 mRNA (A) and protein (B) abundance in tubuli and glomeruli isolated from the kidneys of wild-type mice using sieves; liver is included as a reference. Protein levels were analyzed by densitometry; representative Western blots are shown with pan-actin used as a loading control. Data are mean ± SEM from 3-4 mice.

Figure S3

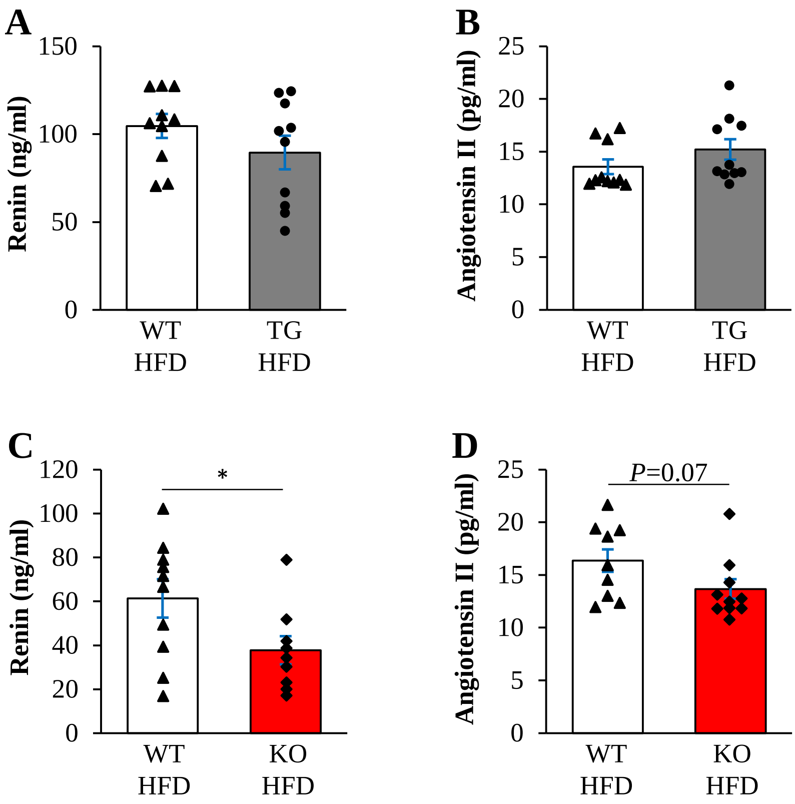

Fig. S3. Depletion of STK25 in high-fat-fed mice affects the renin-angiotensin system. (A-D) Plasma levels of renin (A, C) and angiotensin II (B, D) measured in mice fed a high-fat diet for 20 weeks. Data are mean ± SEM from 9-10 mice per group. HFD, high-fat diet; KO, knockout; TG, transgenic; WT, wild-type. **P*<0.05 by a two-sample Student's *t* test

Figure S4

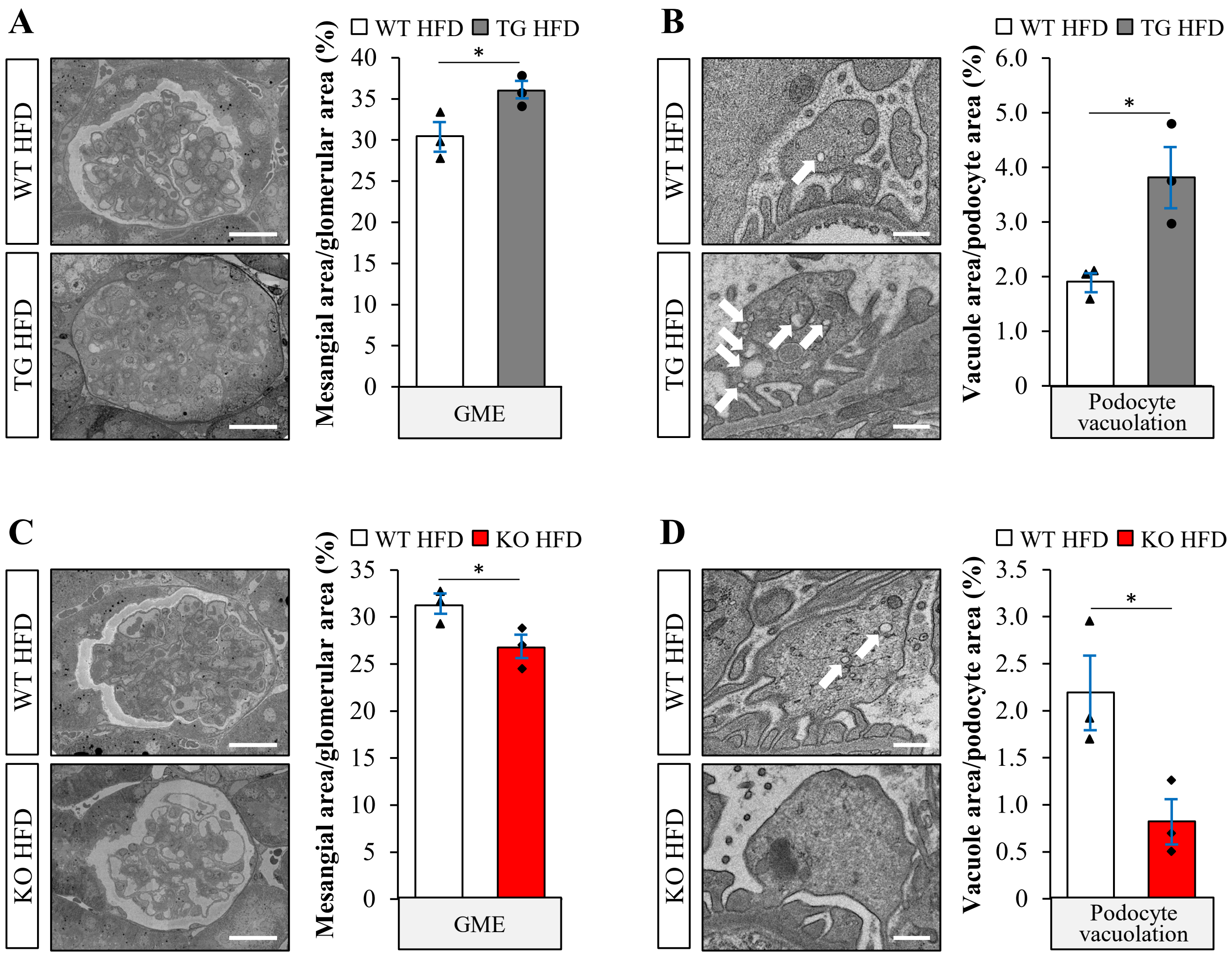

Fig. S4. Electron microscopy analysis of GME and podocyte vacuolation in high-fat-fed mice. (A, C) Representative SEM images of mesangial expansion in glomeruli. Quantification of the mesangial area. (B, D) Representative TEM images of vacuoles (white arrows) in podocytes. Quantification of the vacuole area. In (A, C), the scale bars represent 20 µm; in (B, D), the scale bars represent 500 nm. Data are mean ± SEM from 3 mice per group [5 glomeruli/mouse in (A, C); 22-34 podocytes/mouse in (B, D)]. HFD, high-fat diet; KO, knockout; TG, transgenic; WT, wild-type. *$P \leq 0.05$ by a two-sample Student's *t* test

Figure S5

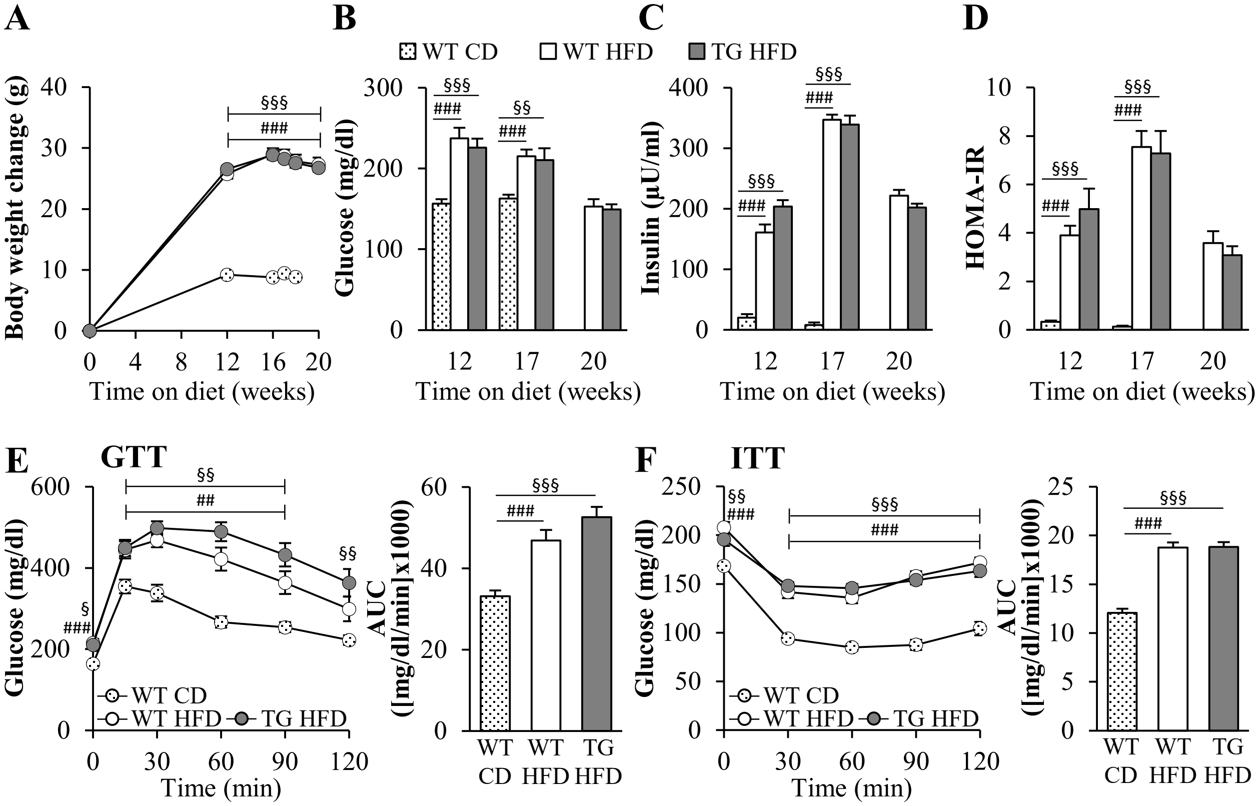

Fig. S5. STK25 overexpression in high-fat-fed mice has no effect on body weight or glucose and insulin homeostasis. (A-D) Body weight change (A), fasting levels of blood glucose (B) and plasma insulin (C); HOMA-IR was calculated using the equation [fasting glucose (mg/dl) x fasting insulin (ng/ml)]/405 (D). The number of weeks on high-fat diet is shown for each measurement. (E-F) Intraperitoneal GTT (E) and ITT (F) after 17 and 19 weeks on a high-fat diet, respectively; the area under the curve for each test is shown. Age-matched chow-fed lean control mice of the same genetic background from our previous study (33) are included as reference. Data are mean ± SEM from 8-14 mice per group. AUC, area under the curve; CD, chow diet; HFD, high-fat diet; TG, transgenic; WT, wild-type. $^{\#\#}P$<0.01, $^{\#\#\#}P$<0.001 for wild-type mice fed high-fat *vs.* chow diet; $^{\S}P$<0.05, $^{\S\S}P$<0.01, $^{\S\S\S}P$<0.001 for wild-type mice fed chow diet *vs.* transgenic mice fed high-fed diet. One-way ANOVA followed by a two-sample Student's *t* test was performed

Figure S6

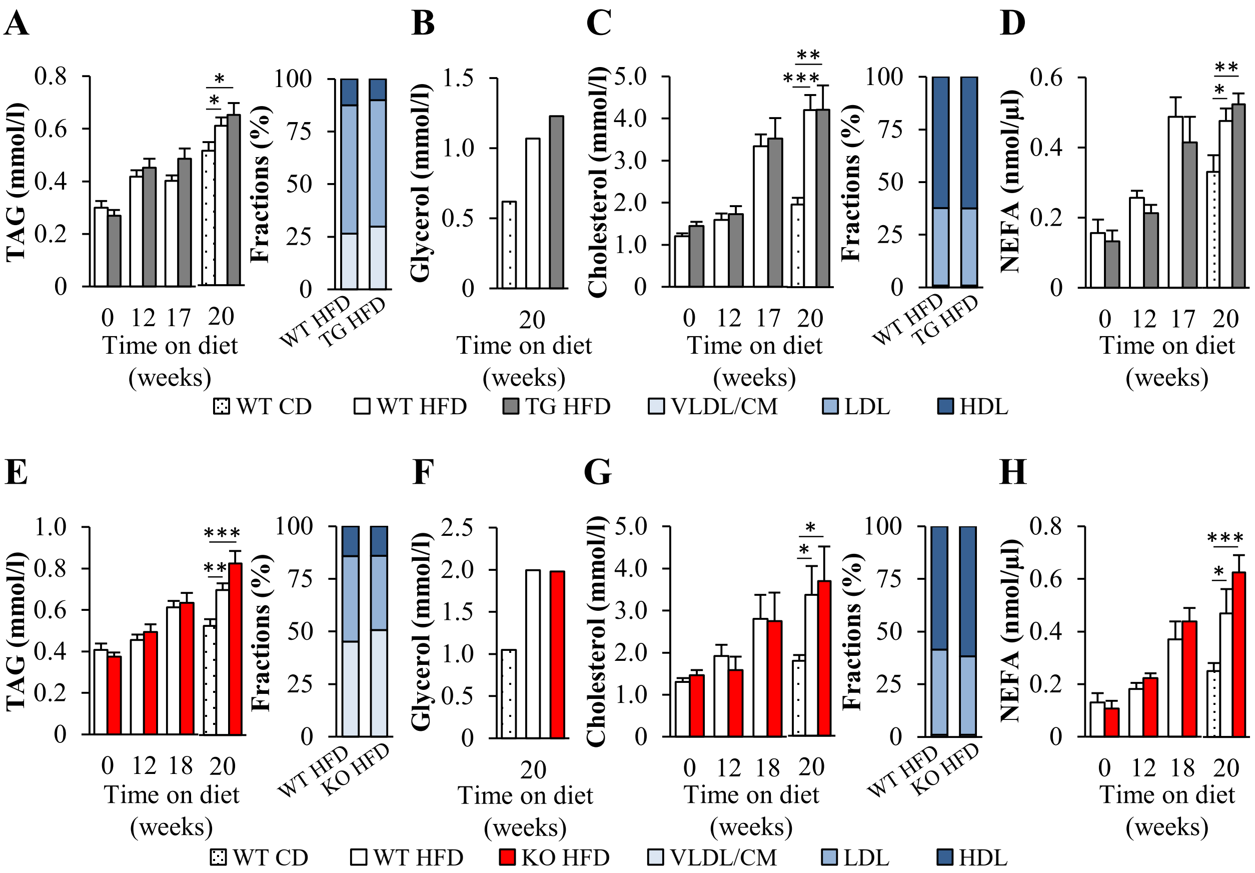

Fig. S6. STK25 does not affect plasma lipid levels in high-fat-fed mice. Fasting plasma concentrations of total TAG and its distribution across the lipoprotein fractions (A, E), glycerol (B, F), total cholesterol and its distribution across the lipoprotein fractions (C, G), and free fatty acids (D, H). The number of weeks on high-fat diet is shown for each measurement, except for lipoprotein fractioning, which is performed on plasma samples from mice fed a high-fat diet for 20 weeks. Age-matched chow-fed lean control mice of the same genetic background are included as reference. Total TAG measurement presented has a limitation of reflecting the sum of TAG and glycerol. Data are mean ± SEM from 7-8 mice per group, except for lipoprotein fractioning and glycerol, which are performed on pooled plasma samples from 8 mice per group. CD, chow diet; CM, chylomicron; HDL, high-density lipoprotein; HFD, high-fat diet; KO, knockout; LDL, low-density lipoprotein; NEFA, non-esterified free fatty acid; TG, transgenic; WT, wild-type. *$P\leq0.05$, **$P<0.01$, ***$P<0.001$ by one-way ANOVA followed by a two-sample Student's *t* test

Figure S7

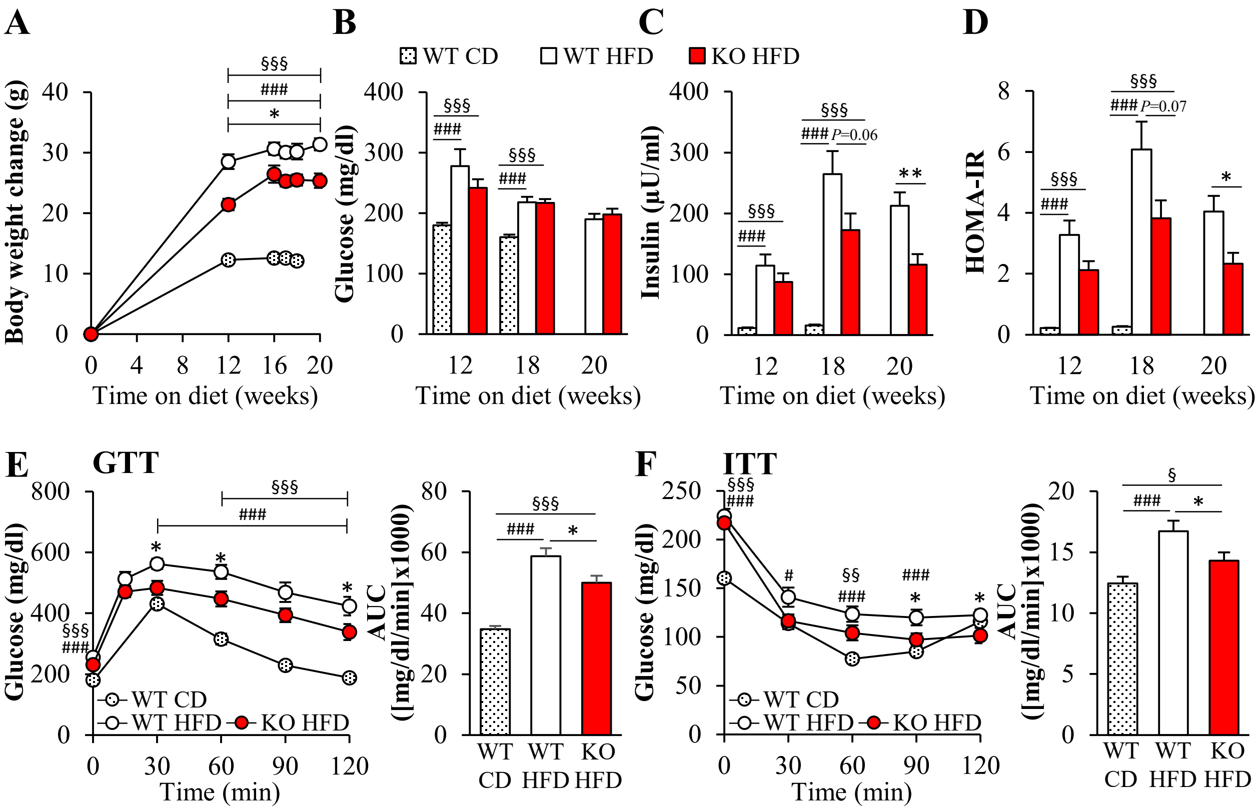

Fig. S7. Knockdown of STK25 in high-fat-fed mice improves whole-body glucose tolerance and insulin sensitivity. (A-D) Body weight change (A), fasting levels of blood glucose (B) and plasma insulin (C); HOMA-IR was calculated using the equation [fasting glucose (mg/dl) x fasting insulin (ng/ml)]/405 (D). The number of weeks on high-fat diet is shown for each measurement. (E-F) Intraperitoneal GTT (E) and ITT (F) after 17 and 19 weeks on a high-fat diet, respectively; the area under the curve for each test is shown. Age-matched chow-fed lean control mice of the same genetic background from our previous study (10) are included as reference. Data are mean ± SEM from 7-13 mice per group. AUC, area under the curve; CD, chow diet; HFD, high-fat diet; KO, knockout; WT, wild-type. $^{*}P<0.05$, $^{**}P<0.01$ for wild-type *vs.* knockout mice fed high-fat diet; $^{\#\#\#}P<0.001$ for wild-type mice fed high-fat *vs.* chow diet; $^{\S}P<0.05$, $^{\S\S}P<0.01$, $^{\S\S\S}P<0.001$ for wild-type mice fed chow diet *vs.* knockout mice fed high-fed diet. One-way ANOVA followed by a two-sample Student's *t* test was performed

Figure S8

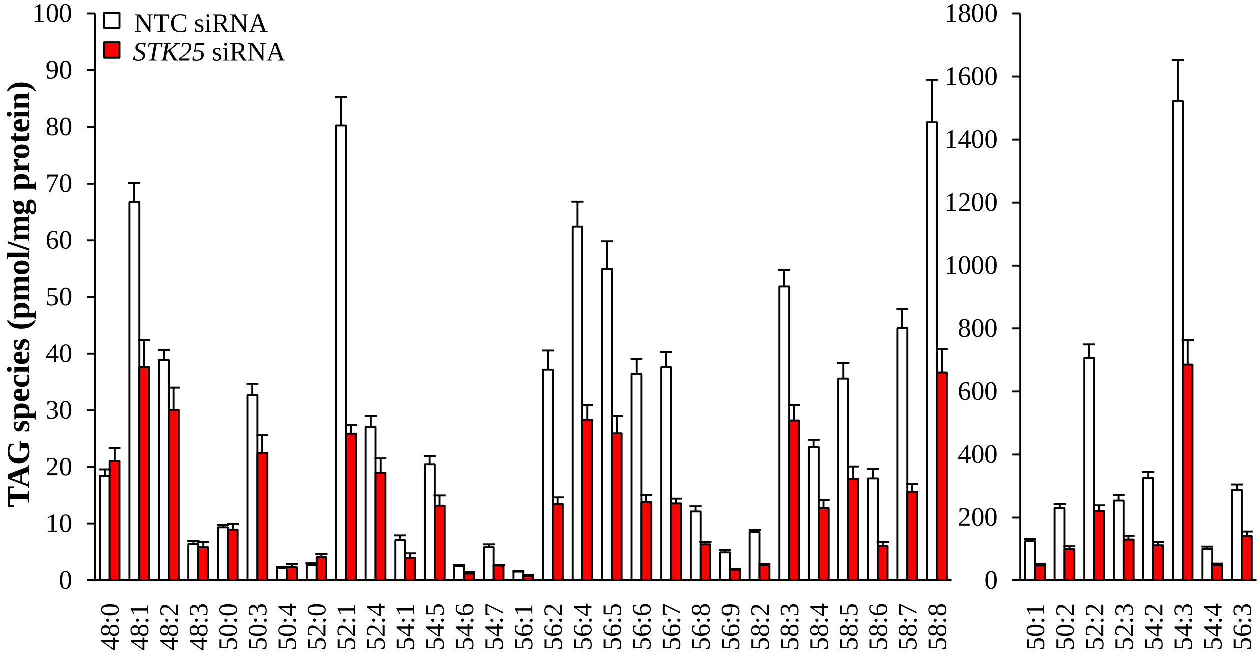

Fig. S8. Silencing of STK25 in HEK293 cells decreases the levels of all the main TAG species. HEK293 cells were transfected with *STK25* siRNA or NTC siRNA and challenged with oleic acid for 48 hours. The TAG species are expressed with total number of carbons and total number of double bonds in the fatty acid moieties. As an example, all TAG species with a total number of 54 carbons and 3 double bonds are written as TAG 54:3. Data are mean ± SEM from 7-8 wells per group. For statistical significances, see Table S1.

Figure S9

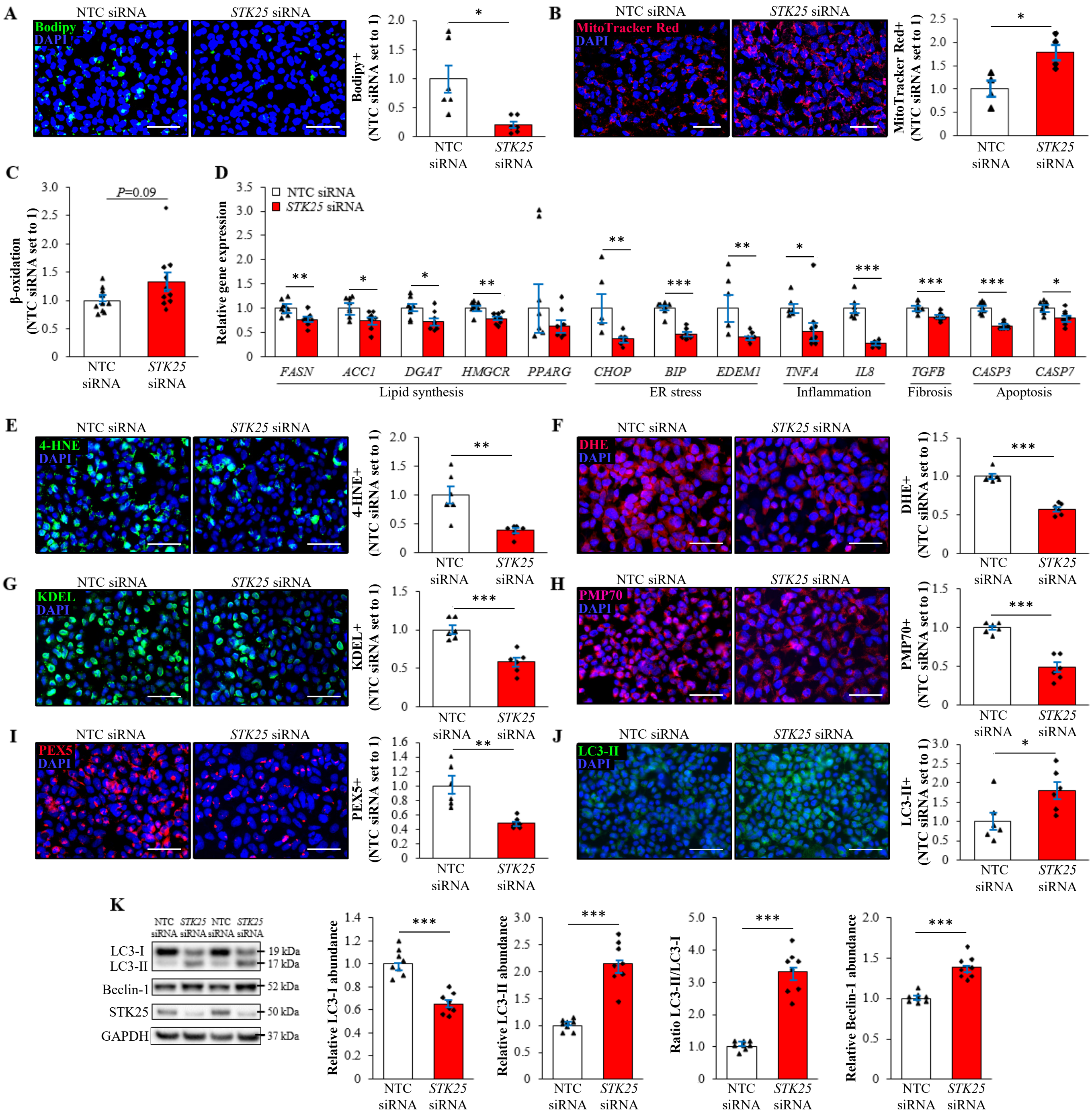

Fig. S9. Silencing of STK25 protects HEK293 cells cultured without oleate supplementation against ectopic lipid storage and oxidative and ER stress, and activates autophagy. HEK293 cells were transfected with *STK25* siRNA or NTC siRNA and maintained under basal culture conditions. (A-B, F) Representative images of cells stained with Bodipy (green; A), MitoTracker Red (red, B), or DHE (red, F); nuclei stained with DAPI (blue). Quantification of the staining. (C) Oxidation of radiolabeled palmitate. (D) Measurement of the mRNA expression of selected markers. The gene functions are indicated at the bottom. (E, G-J) Representative images of cells processed for immunofluorescence with anti-4-HNE (green; E), anti-KDEL (green; G), anti-PMP70 (red; H), anti-PEX5 (red; I), or anti-LC3

(green, J) antibodies; nuclei stained with DAPI (blue). Quantification of the staining. (K) Cell lysates were analyzed by Western blot using antibodies specific for LC3, Beclin-1, and STK25. Protein levels were analyzed by densitometry; representative Western blots are shown with GAPDH used as a loading control. The scale bars represent 40 µm. Data are mean ± SEM from 4-10 wells per group. *$P<0.05$, **$P<0.01$, ***$P<0.001$ by a two-sample Student's *t* test

Figure S10

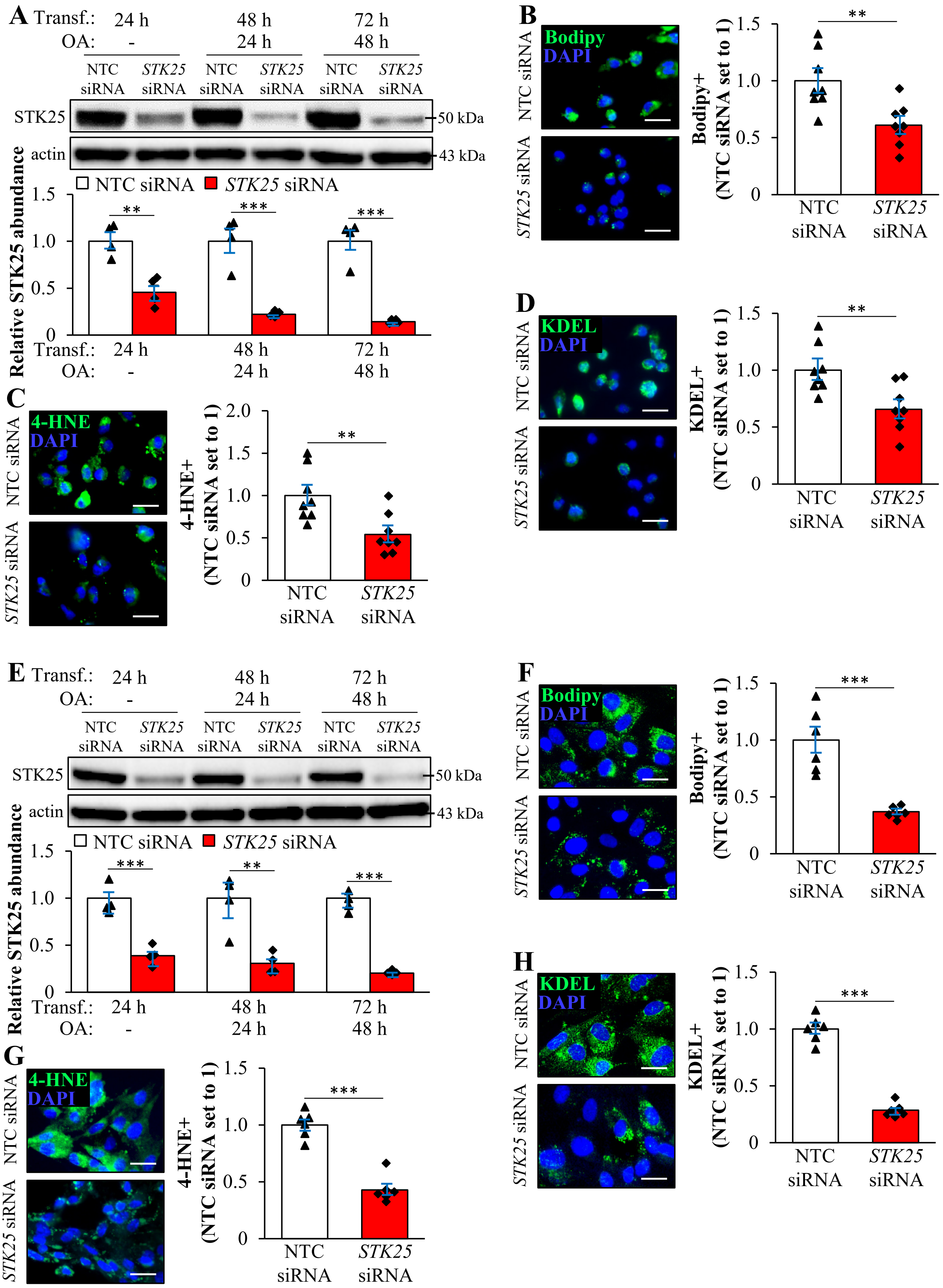
A
Transf.: 24 h 48 h 72 h
OA: - 24 h 48 h
NTC siRNA
STK25 siRNA
STK25
actin
50 kDa
43 kDa
NTC siRNA
STK25 siRNA
Relative STK25 abundance
B
Bodipy
DAPI
Bodipy+ (NTC siRNA set to 1)
C
4-HNE
DAPI
4-HNE+ (NTC siRNA set to 1)
D
KDEL
DAPI
KDEL+ (NTC siRNA set to 1)
E
F
G
H

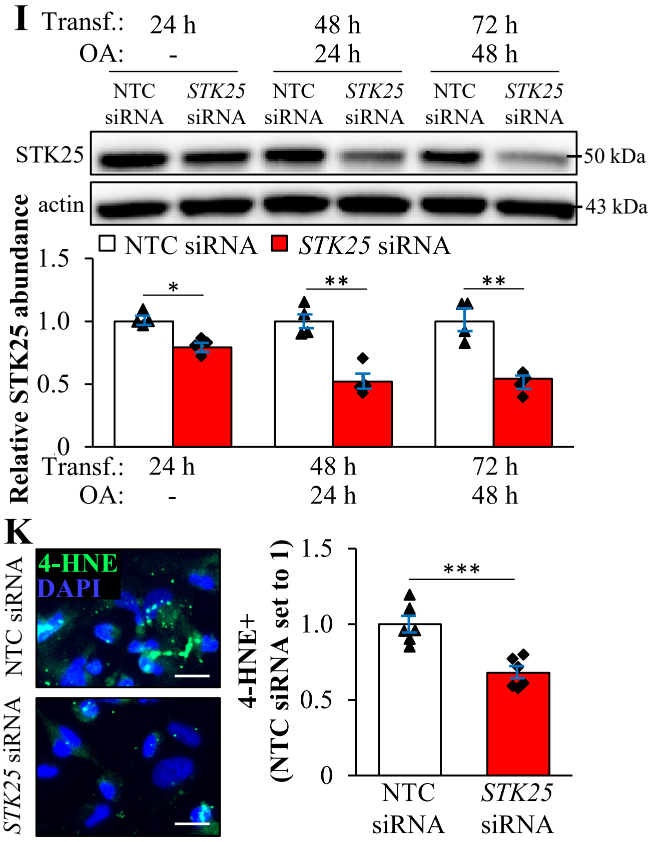

Fig. S10. Silencing of STK25 protects human proximal tubular (HK-2) cells, mesangial cells, and podocytes against lipid deposition as well as oxidative and ER stress. HK-2 (A-D), mesangial cells (E-H), and podocytes (I-L) were transfected with *STK25* siRNA or NTC siRNA and challenged with oleic acid for 48 hours. (A, E, I) STK25 protein abundance. Protein levels were analyzed by densitometry; representative Western blots are shown with pan-actin used as a loading control. (B-D, F-H, J-L) Representative images of cells stained with Bodipy (green; B, F, J), or processed for immunofluorescence with anti-4-HNE (green; C, G, K) or anti-KDEL (green; D, H, L) antibodies; nuclei stained with DAPI (blue). Quantification of the staining. The scale bars represent 20 µm. Data are mean ± SEM from 4-8 wells per group. OA, oleic acid; Transf., transfection. **P*<0.05, ***P*<0.01, ****P*<0.001 by a two-sample Student's *t* test

Figure S11

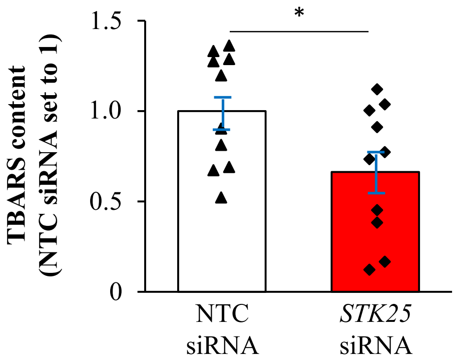

Fig. S11. Silencing of STK25 protects HEK293 cells against oxidative damage. HEK293 cells were transfected with *STK25* siRNA or NTC siRNA and challenged with oleic acid for 48 hours. TBARS content was measured in cell extracts. Data are mean ± SEM from 10 wells per group. **P*<0.05 by a two-sample Student's *t* test

Figure S12

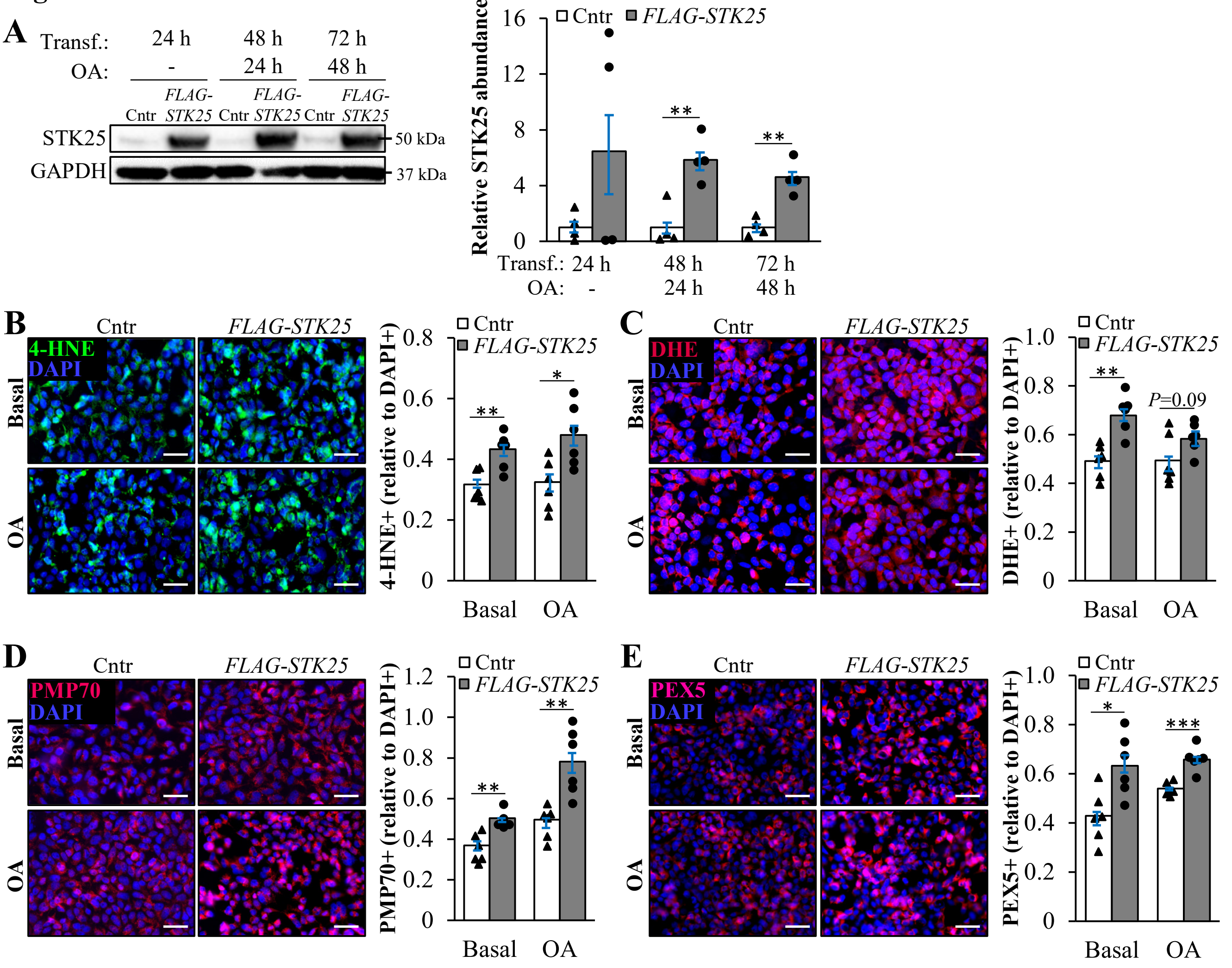

Fig. S12. Overexpression of STK25 in HEK293 cells aggravates oxidative stress and enhances peroxisomal biogenesis, both with and without oleate challenge. HEK293 cells were transfected with *FLAG*-tagged *STK25* expression plasmid or empty control plasmid (mock) and cultured with or without oleate supplementation for 48 hours. (A) STK25 protein abundance. Protein levels were analyzed by densitometry; representative Western blots are shown with GAPDH used as a loading control. (B-E) Representative images of cells processed for immunofluorescence with anti-4-HNE (green; B), anti-PMP70 (red; D), or anti-PEX5 (red; E) antibodies, or stained with DHE (red; C); nuclei stained with DAPI (blue). Quantification of the staining. The scale bars represent 40 µm. Data are mean ± SEM from 4-6 wells per group. Cntr, control; OA, oleic acid; Transf., transfection. **P*<0.05, ***P*<0.01, ****P*<0.001 by a two-sample Student's *t* test

Figure S13

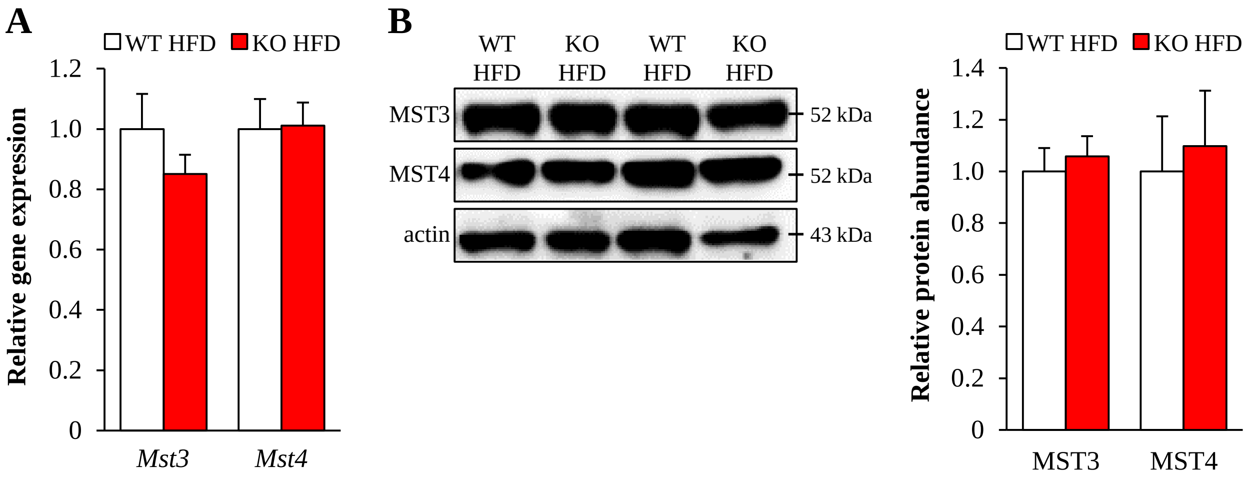

Fig. S13. Depletion of STK25 in high-fat-fed mice is not compensated by increased renal mRNA or protein abundance of MST3 or MST4. (A-B) MST3 and MST4 mRNA (A) and protein (B) abundance in the kidneys from *Stk25* knockout mice and wild-type littermates. Protein levels were analyzed by densitometry; representative Western blots are shown with pan-actin used as a loading control. Data are mean ± SEM from 8-9 mice per group. HFD, high-fat diet; KO, knockout; WT, wild-type.

Table S1. Abundance of individual TAG species in HEK293 cells transfected with *STK25* siRNA or NTC siRNA and challenged with oleic acid for 48 hours

**TAG species**	**Mean ± SEM**		***P* value**
	**NTC siRNA**	***STK25* siRNA**	
48:0	18.4±1.1	21±2.2	0.33
48:1	66.7±3.3	37.6±4.8	**0.0003**
48:2	38.8±1.7	30±3.9	0.07
48:3	6.4±0.5	5.8±0.9	0.61
50:0	9.3±0.4	8.9±0.9	0.74
50:1	124.5±6.7	47.5±4.7	**0.0000003**
50:2	228.8±13.6	98.7±9	**0.000002**
50:3	32.7±1.9	22.4±3.1	**0.02**
50:4	2.1±0.2	2.3±0.5	0.82
52:0	2.6±0.3	4.1±0.5	0.06
52:1	80.2±5	25.8±1.4	**0.0000001**
52:2	706.5±42.5	221.3±16.6	**0.00000005**
52:3	253.3±18	129.4±12.2	**0.00006**
52:4	27.0±1.9	19.0±2.5	**0.03**
54:1	7.1±0.8	3.9±0.7	**0.02**
54:2	324.9±19.1	111.4±9.7	**0.0000001**
54:3	1521.8±131.4	685.4±78.6	**0.00008**
54:4	99.8±7.1	47.8±5.2	**0.00005**
54:5	20.4±1.4	13.2±1.7	**0.009**
54:6	2.5±0.2	1.2±0.2	**0.002**
54:7	5.8±0.5	2.6±0.1	**0.00002**
56:1	1.5±0.1	0.7±0.1	**0.001**
56:2	37.1±3.3	13.4±1.1	**0.00001**
56:3	287.3±16.7	140.7±13.7	**0.00001**
56:4	62.4±4.4	28.3±2.6	**0.00001**
56:5	54.9±4.8	25.9±3.0	**0.0002**
56:6	36.3±2.6	13.8±1.2	**0.000002**
56:7	37.6±2.7	13.6±0.8	**0.0000006**
56:8	12.1±0.8	6.3±0.4	**0.00004**
56:9	4.9±0.3	1.8±0.1	**0.000003**
58:2	8.4±0.4	2.6±0.2	**0.00000001**
58:3	51.8±2.9	28.2±2.7	**0.00005**
58:4	23.5±1.3	12.7±1.4	**0.0001**

TAG species	Mean ± SEM		*P* value
	NTC siRNA	*STK25* siRNA	
58:5	35.5±2.7	17.9±2	**0.0002**
58:6	17.9±1.6	6.1±0.7	**0.00001**
58:7	44.5±3.3	15.5±1.3	**0.000001**
58:8	80.8±7.4	36.6±4.1	**0.0001**

All lipid species are expressed in pmol/mg protein. Data are mean ± SEM from 7-8 wells per group. Bold text indicates significant *P* value.

Table S2. List of antibodies used for Western blot and immunofluorescence analysis

Type	Antibody name and catalogue number	Working dilution	Company
Primary antibody	anti-4-HNE (ab46545)	1:500	Abcam (Cambridge, UK)
	anti-α-SMA (a5694)	1:100	Abcam
	anti-adipophilin (ab181452)	1:500	Abcam
	anti-Beclin-1 (#3495)	1:1000	Cell Signaling Technology (Boston, MA)
	anti-CD68 (ab53444)	1:200	Abcam
	anti-collagen IV (ab6586)	1:500	Abcam
	anti-GAPDH (J0914)	1:1000	Cell Signaling Technology
	anti-JNK (#9252)	1:1000	Cell Signaling Technology
	anti-KDEL (ab176333)	1:1000	Abcam
	anti-LC3 (#2775)	1:200	Cell Signaling Technology
	anti-nephrin (#L1917)	1:100	Santa Cruz Biotechnology (Santa Cruz, CA)
	anti-pan-actin (L3014)	1:500	Santa Cruz Biotechnology
	anti-Pecam (558736)	1:100	BD Biosciences (San Jose, CA)
	anti-PEX5 (PA5-58716)	1:200	Invitrogen (Carlsbad, CA)
	anti-phospho-JNK (#4668)	1:1000	Cell Signaling Technology
	anti-PMP70 (PA1-650)	1:200	Invitrogen
	anti-STK25 (25821-1-AP)	1:200	Proteintech (Chicago, IL)
Secondary antibody	Alexa Fluor-488-labeled anti-mouse IgG (A21202)	1:500	Invitrogen
	Alexa Fluor-488-labeled anti-rabbit IgG (A11008)	1:500	Invitrogen
	Alexa Fluor-594-labeled anti-mouse IgG (A11005)	1:500	Invitrogen
	Alexa Fluor-594-labeled anti-rabbit IgG (A21207)	1:500	Invitrogen
	anti-rabbit IgG (#7074)	1:1000	Cell Signaling Technology
	anti-mouse IgG (#7076)	1:1000	Cell Signaling Technology

Table S3. Differentially represented metabolites in the kidneys from *Stk25* knockout *vs.* wild-type mice fed a high-fat diet

**Metabolite**	***P* value**	**KO/WT**
Threonine	0.006	-1.61
Serotonin	0.01	-1.49
GSH/GSSG	0.01	1.46
Putrescine/ornithine	0.04	1.42
Serine	0.02	-1.41
C0/(C16+C18)	0.0498	1.37
Aspartic acid	0.04	-1.29
Essential amino acids	0.007	-1.23
Methionine	0.02	-1.22
Ornithine	0.04	-1.22
Proline	0.005	-1.19

A ratio of 1.15-fold serves as the threshold for differential regulation. Data are from 6-8 mice per group. KO, knockout; WT, wild-type.
